# An Updated Overview of Magnetic Composites for Water Decontamination

**DOI:** 10.3390/polym16050709

**Published:** 2024-03-05

**Authors:** Adelina-Gabriela Niculescu, Bogdan Mihaiescu, Dan Eduard Mihaiescu, Tony Hadibarata, Alexandru Mihai Grumezescu

**Affiliations:** 1 Research Institute of the University of Bucharest—ICUB, University of Bucharest, 050657 Bucharest, Romania; adelina.niculescu@upb.ro (A.-G.N.); bogdan.mihaiescu@upb.ro (B.M.); 2 Department of Science and Engineering of Oxide Materials and Nanomaterials, University Politehnica of Bucharest, Gh. Polizu St. 1-7, 060042 Bucharest, Romania; hadibarata@curtin.edu.my; 3 Department of Organic Chemistry, Politehnica University of Bucharest, 011061 Bucharest, Romania; danedmih@gmail.com; 4 Environmental Engineering Program, Faculty of Engineering and Science, Curtin University, Miri 98009, Malaysia

**Keywords:** water remediation, magnetic nanoparticles, magnetic composites, composites for water decontamination, magnetic composite adsorbents, dye removal, heavy metal removal, drug removal

## Abstract

Water contamination by harmful organic and inorganic compounds seriously burdens human health and aquatic life. A series of conventional water purification methods can be employed, yet they come with certain disadvantages, including resulting sludge or solid waste, incomplete treatment process, and high costs. To overcome these limitations, attention has been drawn to nanotechnology for fabricating better-performing adsorbents for contaminant removal. In particular, magnetic nanostructures hold promise for water decontamination applications, benefiting from easy removal from aqueous solutions. In this respect, numerous researchers worldwide have reported incorporating magnetic particles into many composite materials. Therefore, this review aims to present the newest advancements in the field of magnetic composites for water decontamination, describing the appealing properties of a series of base materials and including the results of the most recent studies. In more detail, carbon-, polymer-, hydrogel-, aerogel-, silica-, clay-, biochar-, metal–organic framework-, and covalent organic framework-based magnetic composites are overviewed, which have displayed promising adsorption capacity for industrial pollutants.

## 1. Introduction

Only one percent of the water on Earth is usable freshwater, of which about 70% is used in agriculture and other commercial purposes. Over a billion people do not have access to clean freshwater, and this limited resource is becoming scarcer globally due to various factors, including population growth, climate change, deforestation, water pollution, and wasteful water use. Contaminated water by industrial pollutants and pathogens adversely affects human health and the environment. Freshwater contamination can arise from various sources, including sewage, agricultural waste, industrial waste, petroleum slicks, nuclear and thermal pollution, pesticides and fertilizers, the mining industry, and population growth and urbanization [1,2,3].

Latent sources of drinking water still exist in rivers, streams, lakes, and subterranean aquifers. Nonetheless, it is essential to remediate any water obtained from surface sources in order to protect against the risk of ingesting various contaminants [4]. Untreated wastewater often contains hazardous substances that may contaminate land or water where sewage is dumped [5]. Therefore, wastewater treatment and disposal are not only desirable but vital in the current global context.

**Figure 1:**
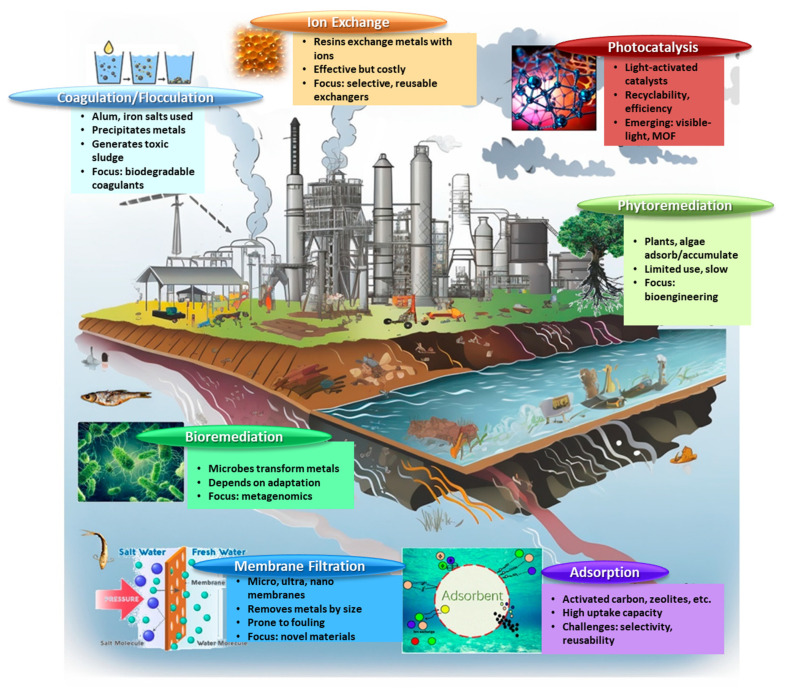
Overview of heavy metal removal methods. Adapted from an open access source [11].

The main treatment process carried out in wastewater treatment plants follows the standard process train: (i) preliminary treatment (involves screening and grit removal using physical methods and setting the stage for primary treatment); (ii) primary treatment (using sedimentation to reduce the organic load in wastewater); (iii) secondary treatment (employing biological methods such as activated sludge that is further treated or used); and (iv) tertiary treatment (encompasses advanced processes like filtration, chemical coagulation, flocculation, flotation, nutrient removal, adsorption, advanced oxidation processes (AOPs), ion exchange, membrane processes (reverse osmosis, nanofiltration, etc.), constructed wetlands, electrocoagulation, and disinfection) [6,7,8,9,10]. When it comes to wastewater, tertiary treatment (Figure 1) is the one referred to as water decontamination in most studies.

The effectiveness of conventional techniques (e.g., sedimentation, chemical precipitation, solvent extraction, ion exchange, and membrane separation) in eliminating heavy metals from water and wastewater is widely recognized. However, these techniques have certain drawbacks, including the need for expensive equipment, constant monitoring, a considerable volume of sludge or solid wastes, chemical reagents, and an incomplete treatment process [12]. Scientists worldwide have become increasingly interested in finding affordable and environmentally friendly methods of disinfecting water [13]. Particular attention has been drawn to adsorption, a method successfully employed for pollutant removal in water decontamination processes. This approach has an easy-to-understand design and functioning, and it is reasonably priced. Many materials, such as carbon-based materials, various synthetic porous composites, naturally occurring inorganic minerals like clay or zeolites, and functionalized natural and synthetic polymers, have been rendered effective at removing organic and inorganic contaminants from polluted water samples [12].

Recent advancements in nanotechnology can also be exploited for water decontamination applications, with a special focus on multifunctional nanomaterials made from nontoxic and cheap precursors. In particular, magnetic nanostructures hold promise for their use in the development of performant adsorbents of heavy metals and organic pollutants [12,13]. In this respect, the inclusion of intrinsic magnetic metal nanoparticles, such as iron, cobalt, and nickel, into various composites has been extensively explored in recent research [14]. When it comes to the separation and recovery of nanomaterials, magnetic separation is an eco-friendly option compared to filtration or centrifugation since it uses lower amounts of solvents and auxiliaries, takes less time to operate, and is more economical. Hence, magnetic adsorbents have emerged as a new class of materials for decontamination procedures [15]. Thus, numerous water decontamination studies have reported the successful utilization of magnetic nanoparticles in combination with a wide range of materials, extending the knowledge in the field and offering encouraging perspectives for environmental applications.

In this context, this paper aims to briefly present magnetic nanoparticles, providing evidence on their properties of interest for water decontamination applications, and further overviews the recent developments in magnetic composites for pollutant removal from aqueous samples. Several literature reviews have already been published in the field, encompassing certain aspects related to magnetic adsorbents for water depollution [16,17,18,19,20,21,22]. Nonetheless, they were either issued a few years ago or addressed only one category of composites/contaminants. Given the effervescence of the field, an updated, extensive perspective is needed to help researchers optimize current materials and implement better-performing magnetic adsorbents in real-life applications.

Thus, herein, a broader approach is adopted to present the most up-to-date literature with the newest advancements in this interdisciplinary domain. Specifically, the latest studies (published in the last 7 years) are included and comprehensively discussed in several sections according to the base material. Carbon-, polymer-, hydrogel-, silica-, aerogel-, biochar-, clay-, covalent organic framework-, and metal–organic framework-based magnetic composites are considered in this review. Moreover, recent studies corresponding to each category are tabulated, offering a clear image of their most important properties and utility in removing toxic metals, dyes, pesticides, drugs, oils, organic solvents, and other harmful compounds from contaminated water samples.

## 2. Magnetic Nanoparticles

**Figure 2:**
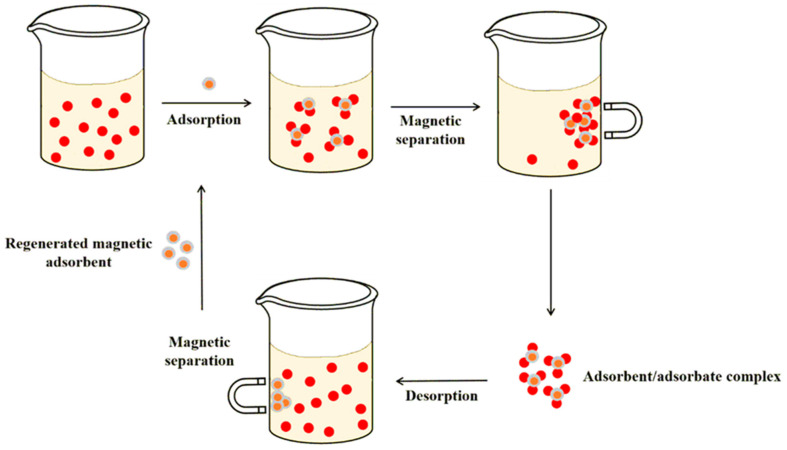
Schematic representation of liquid-phase adsorption employing magnetic nanoadsorbents. Reprinted from an open access source [24].

In addition to established methods for remediating contaminated wastewater, novel approaches that employ magnet-sensitive materials are progressively gaining traction [23]. Integrating composite adsorbents with a magnetic component enables the facile separation of adsorbate complexes from aqueous solutions and easy regeneration and reuse of the material for future decontamination cycles by simply applying an external magnetic field [24] (Figure 2). In more detail, magnetic nanocomposites are dispersed in a sample solution to adsorb contaminants through specific interactions (e.g., electron transfer, chemical bond formation, van der Waals forces, electrostatic interactions, H bonds, and π–π bonds). After adsorption is accomplished, the magnetic material loaded with the captured pollutants is separated from the treated water with an external magnet. Then, with the aid of a desorption solvent, the contaminants are removed from the magnetic composites, and the adsorbent is regenerated (through chemical treatment) and can be used again for decontamination of other water samples. Hence, magnetic solid-phase extraction offers a simple, rapid, eco-friendly, and economical possibility for recycling magnetic composites involved in environmental purification [24,25,26,27].

The selection of magnetic material is a critical step in the magnetic separation process. Different types of magnetic particles have lately been produced, and they have shown considerable promise for separation in water treatment applications [28]. Fe, Ni, and Co are metals recognized for their appealing intrinsic magnetic properties [23]. Exploring their combined potential [29,30,31,32,33,34] or using them in association with different materials to develop valuable composites [35,36,37,38] have been established as promising solutions for water decontamination.

Additionally, different iron oxides have been considered in numerous studies. Particularly, magnetite (Fe_3_O_4_) and maghemite (γ-Fe_2_O_3_) have shown promise in their use for the development of advanced composite materials [23,39,40]. Both maghemite and magnetite have a spinel crystal structure, while the latter contains both divalent and trivalent iron cations. In maghemite, all iron cations are trivalent, and the presence of cation vacancies ensures the cell’s charge neutrality [41]. These iron oxides benefit from easy synthesis routes, eco-friendliness, and high saturation magnetization [15,42]. Magnetite is the most magnetic naturally occurring mineral, having an 84 emu/g saturation magnetization and small magnetic anisotropy at room temperature [41]. Moreover, magnetite nanoparticles exhibit thermal, chemical, and colloidal stability; dispersibility; and functionalization possibility—advantageous features that extend their versatility [42]. Other ferrites that showed good promise in environmental purification applications include barium hexaferrite (BaFe_12_O_19_; excellent electrical, magnetic, optical and photocatalytic properties; remarkable stability; and reusability) [43,44,45], strontium hexaferrite (SrFe_12_O_19_; high saturation magnetization, great stability, and excellent photocatalytic properties) [46], and lead hexaferrite (PbFe_12_O_19_; high saturation magnetization, coercivity, catalytic activity, and stability) [47,48].

Nonetheless, given their elevated surface energy, bare magnetic iron oxide nanoparticles are susceptible to coaggregation and oxidation/dissolution, particularly in acidic solutions. Their chemical stability can be compromised even under environmental conditions, constraining their potential for large-scale implementation. Therefore, improved solutions have been generated by using functionalized magnetic nanoparticles and magnetic nanocomposites, featuring novel surface properties and structures to address the shortcomings of magnetic nanoparticles, increase their number of active sites, enhance their stability in aqueous environments, and increase separation efficiency [15,39,42,49,50].

## 3. Magnetic Composites for Water Decontamination

**Figure 3:**
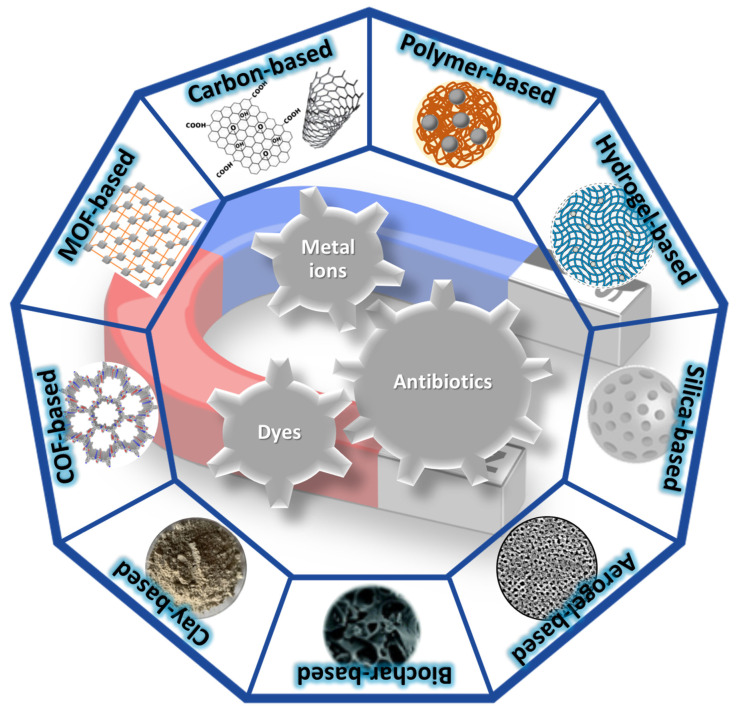
Schematic representation of magnetic composite materials for the removal of organic and inorganic pollutants from contaminated water.

Given the appealing properties of magnetic nanoparticles, numerous magnetic composite materials (Figure 3) have been developed with the purpose of providing performant water decontamination solutions. The illustrated categories of materials are detailed in subsequent subsections, including relevant examples of magnetic adsorbents from recent studies in the literature (published since 2018).

### 3.1. Carbon-Based Composites

Carbon structures are among the most studied materials due to their appealing properties, such as mechanical strength, chemical stability, anisotropy, and high conductivity [22]. Carbon-based materials, including activated carbon, carbon nanotubes, graphene oxide, graphitized carbon black, and porous carbon, have been used for pollutant adsorption. What renders them suitable for application in water decontamination processes is the versatility of their interactions with targeted pollutants, which can be electrostatic, hydrophobic, or π–π interactions [15].

Moreover, combining carbon nanostructures with magnetic nanoparticles leads to synergic composites with exceptional magnetic features [22]. Magnetic nanohybrids are particularly relevant due to their facile separation from aqueous samples after the adsorption of pollutants. In addition to the inherent benefits of magnetic separation (e.g., eco-friendliness, easy operation, and cost-effectiveness), this method facilitates magnetic carbon-based nanocomposite regeneration and reuse for several adsorption/desorption cycles of wastewater treatment [20,22].

**Figure 4:**
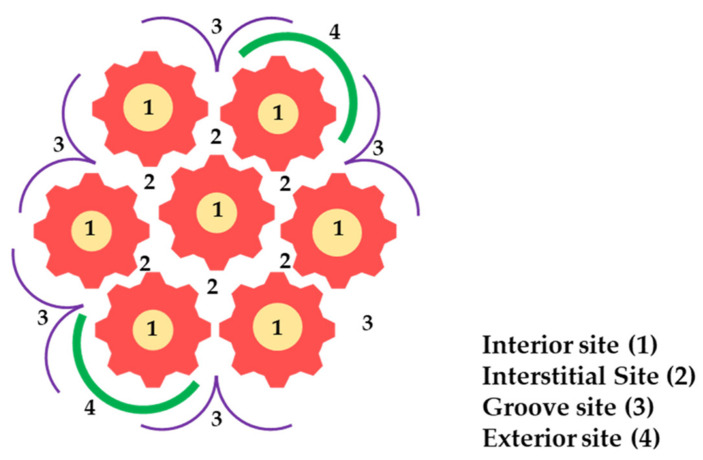
Adsorption sites of CNTs for water pollutants. Reprinted from an open access source [53].

Among carbon-based materials, carbon nanotubes (CNTs) have received the most scientific interest in recent years. Given their unique morphology, surface chemistry, and chemical and physical interactions with organic and inorganic compounds, CNTs have been extensively employed in water remediation processes. Thus, CNTs have surfaced as a profitable, efficient, and environmentally sustainable substitute for conventional water treatments, being involved in the elimination of a wide range of water contaminants [51]. Having numerous adsorption sites (Figure 4), CNTs exhibit the ability to remove both organic and inorganic pollutants from aqueous samples. Interstitial and groove sites initiate the adsorption process, which is followed by pollutant adsorption on exterior walls and the accumulation of captured molecules within interior channels [52]. Interstitial sites are particularly fitted for trapping small contaminants depending upon the form of the nanotube, whereas exterior sites and grooves present enough space for the adsorption of both inorganic and organic contaminants [53].

In recent years, CNTs have been investigated as promising solutions for heavy metal removal, and they have been confirmed as strong adsorbents for metal ion adsorption, including water decontamination from Ni^2+^, Sr^2+^, Pb^2+^, Zn^2+^, Cu^2+^, Cd^2+^, Co^2+^, and Cr^2+^ [53,54,55,56]. However, in comparison, much more extensive progress has been made lately in designing CNT-based adsorbents for organic pollutant removal, with numerous recent studies reporting the encouraging outcomes obtained when associating these carbon-based nanomaterials with magnetic components.

An impressive number of papers have revealed the potential of magnetic CNT-based composites in extracting organic dyes from aqueous samples, such as methylene blue [32,36,37,57,58,59,60,61,62,63,64,65,66,67], methyl orange [32,36,37,67], malachite green [36,59], Congo red [32,36], rhodamine B [32,36,63,68,69,70,71], crystal violet [63], acid fuchsin [59], neutral red [37], basic violet [72], azure II [73], acid orange [72], methyl violet [74], patent blue V [75], reactive black 5 [35], reactive violet 2 [76], Nile blue [74], alizarin red S [77], and Maxilon blue 5G [78]. Extensive research has also been carried out to verify the ability of magnetic CNT-based composites to remove drugs/active pharmaceutical ingredients from contaminated water, with encouraging studies being reported for the elimination of carbamazepine [79,80,81], ketoprofen [82], estriol [82], tetracycline and its derivatives [70,83,84,85,86,87], sulfamethoxazole [80,88,89,90], phenytoin [81], oxcarbazepine [81], diclofenac [91], caffeine [92], paracetamol [92,93,94], and ibuprofen [94]. Other organic substances that can be successfully removed from water with the use of magnetic CNT-based materials include herbicides (e.g., metolachlor [82], metribuzin [95], diquatdibromide [96]), insecticides (e.g., profenofos, triazophos, diazinon, phosalone, methidathion, ethoprop, sulfotep, and isazofos) [97], pesticides (e.g., pentachlorophenol) [33], agricultural nutrients (e.g., humic acid) [98], chemical intermediates used in industrial production (e.g., bisphenol A [82,99,100], bisphenol AF [95], perfluoroalkyl carboxylic acids [101], perfluoroalkyl sulfonic acids [101], toluene [102], m-cresol [103], esters [104], and phenol derivatives [57,67,105,106,107,108,109,110,111]), and ingredients from cosmetics (e.g., tonalide [82], triclosan [82], butylparaben [104], methylparaben [104], and phthalates [104,112]).

For a better presentation of the plethora of studies in the field, Table 1 summarizes the magnetic CNT-based composites, some of their relevant properties, and targeted organic pollutants.

Other highly exploited carbon-based materials are graphene and its derivatives, as they present certain appealing features (e.g., excellent mechanical properties, unique pore structure with large specific surface area, and ionic molecular sieving separation capabilities) [113]. Particularly, graphene oxide has attracted interest for its applications related to water decontamination, for both organic and inorganic pollutant removal. In combination with magnetic materials, graphene oxide adsorbents benefit from unique physicochemical characteristics, such as desirable magnetic features, surface active sites, tunable dimension morphology, ease of modification/functionalization, and enhanced chemical stability [15,114]. Adding these advantages to their affordability and magnetic separation possibility, magnetic graphene oxide-based composites have been investigated for the removal of many organic pollutants (e.g., dyes [65,115,116,117,118,119], drugs [120,121,122,123], and pesticides [124]) (Table 2) and heavy metals (e.g., Pb^2+^ [125,126,127,128,129,130], Cr^3+^ [127,129,131], Cr^6+^ [131,132,133,134], Hg^2+^ [119,135], Cu^2+^ [61,127,129,134], Zn^2+^ [127], Ni^2+^ [127,129], As^5+^ [119,136], Cd^2+^ [128,129], Co^2+^ [129], and Ag^+^ [129]) (Table 3) [21,114,137].

**Table 1:** Magnetic carbon nanotube-based adsorbents for organic pollutant removal from contaminated water.

Adsorbent	Surface Area (m^2^ g^−1^)	Magnetic Saturation (emu g^−1^)	Pollutant	Adsorption Capacity (mg g^−1^)	References
Carbon dot- and magnetite-modified magnetic CNTs	184	5.6	Carbamazepine	65	[79]
Magnetic CNTs	-	-	Metolachlor Bisphenol A Tonalide Triclosan Ketoprofen Estriol	20.53 28.55 27.32 19.68 26.67 18.24	[82]
CNT-incorporated MIL-88B-Fe	118.10	-	Phenol	-	[105]
Nickel nanoparticles encapsulated in porous carbon/CNT hybrids	999	3.66	Malachite green Congo red Rhodamine B Methylene blue Methyl orange	898 818 395 312 271	[36]
Nickel nanoparticle-decorated graphene oxide CNTs	71.7	-	Rhodamine B	41.5	[68]
Magnetic CNT-reduced graphene oxide–silver nanocomposite	-	6.8	Methylene blue 4-Nitrophenol	-	[57]
CNTs/Fe@C hybrids	186.3	3.64	Methylene blue Methyl orange Neutral red	132.58 16.53 98.81	[37]
Magnetic multiwalled CNT	108.1	-	Patent blue V	-	[75]
Magnetic nanocomposite cobalt multiwalled CNT	87.1457	-	Methylene blue	324.34	[58]
N-doped bamboo-like CNT encapsulated with Fe nanoparticles supported by biochar	194.8 at 700 °C, 225.4 at 800 °C 205.6 at 900 °C	-	Rhodamine B	-	[69]
Core–shell ZIF-67/ZIF-8-derived sea urchin-like cobalt/nitrogen Co-doped CNT hollow frameworks	269.79	41.88	Methyl blue Acid fuchsin Malachite green	8862.5 8032.5 6043.2	[59]
Graphene-templated zeolite-imidazolate framework (ZIF-67) derived, Co nanoparticle embedded, nitrogen-doped CNT	389	-	Reactive black 5	-	[35]
Magnetic fluorinated CNT	-	47.7	Perfluoroalkyl carboxylic acids Perfluoroalkyl sulfonic acids	-	[101]
Magnetic titanium nanotube–CNT nanocomposite	574.1	25.55	Bisphenol A	-	[99]
Aminated MIL-53(Al)-functionalized CNT	811	-	Bisphenol AF Metribuzin	274 213	[95]
Magnetic and N-doped CNT with cobalt encapsulation	125.5	1.61	Oxalic acid	5	[138]
Multiwalled CNT-functionalized MIL-53(Fe)	60.17	-	Tetracycline hydrochloride Oxytetracycline hydrochloride Chlortetracycline hydrochloride	364.37 325.59 180.68	[83]
Multiwalled CNT–amino-functionalized MIL-53(Fe) composites	-	-	Tetracycline hydrochloride Chlortetracycline hydrochloride	368.49 254.04	[84]
MIL-100(Fe)-CNT	1228	-	Oxytetracycline	429	[85]
Magnetic nanomaterial of surface oxidized nano-cobalt wrapped by nitrogen-doped CNTs	243.63–277.62	4.64	Tetracycline Rhodamine B	679.56 385.60	[70]
Nitrogen-doped CNTs with encapsulated Fe_3_C nanoparticles	-	12.6	Sulfamethoxazole	-	[88]
Metal–organic framework ZIF-8/magnetic multiwalled CNTs	127.95	53.56	Profenofos Triazophos Diazinon Phosalone Methidathion Ethoprop Sulfotep Isazofos	3.89 3.12 2.59 3.80 2.34 2.18 2.84 3.00	[97]
Iron-loaded CNT microfibrous composite	-	-	M-cresol	-	[103]
Magnetic nitrogen-doped CNT cages	-	-	Okadaic acid	897.8 µg g^−1^	[139]
Amino-functionalized multiwalled CNTs embedded with magnetic nanoparticles	202.4	-	Methylene blue	178.5	[60]
Magnetic CNT–TiO_2_ composite	-	-	Carbamazepine and Sulfamethoxazole	1.4	[80]
Magnetic CNT composites	-	35.8	Methylene blue	-	[61]
Maghemite nanocrystals decorated multiwalled CNTs	-	-	Methylene blue	59.4	[62]
Magnetic multiwalled CNTs	-	-	Crystal violet Methylene blue Rhodamine B	287 302 231	[63]
Magnetite/multiwalled CNTs	-	51.144	Reactive violet 2	52.356	[76]
Polyethyleneimine (PEI)-functionalized magnetic CNTs	127.93	27.3	Alizarin Red S	196.08	[77]
Magnetic multiwalled CNTs modified with chitosan biopolymers	-	-	Bisphenol A	46.2	[100]
Polydopamine-coated Fe_3_O_4_ nanoparticles with multiwalled CNTs	-	37.96	Phenytoin Oxcarbazepine Carbamazepine	-	[81]
Magnetic multitemplate molecularly imprinted polymer@MWCNTs	-	25.6	Diethyl phthalate Dimethyl phthalate Dibutyl phthalate	1.38 0.95 7.09	[112]
Magnetic single-wall CNTs	-	-	Diclofenac		[91]
Oxidized multiwalled CNT-Fe_3_O_4_	169.0	-	Diquatdibromide herbicide	20.9	[96]
Oxidized multiwalled CNT–κ-carrageenan–Fe_3_O_4_	142.2	-	Diquatdibromide herbicide	10.7	[96]
Magnetic multiwalled CNTs modified with polyaluminum chloride	215.90	7.98	Humic acid	-	[98]
Multiwalled carbon nanotube-modified magnetic polyamidoamine dendrimers	-	47.71	Heterocyclic aromatic hydrocarbons	-	[140]
Single-wall CNTs and magnetic nanoparticles	284.21	-	Toluene	49.8	[102]
Magnetic multiwalled CNTs/cerium dioxide nanocomposite	-	8.22	Methylene blue		[64]
Multiwalled CNT-based Fe_3_O_4_	-	-	Maxilon Blue 5G	-	[78]
Multiwalled CNT–NiFe_2_O_4_ composite	-	33.1	Sulfamethoxazole	-	[89]
Cobalt ferrite–CNT nanocomposites	-	56	Methylene blue	8.5178	[65]
NiFe_2_O_4_/MWCNTs/ZnO hybrid nanocomposite	-	17.021	Methylene blue	-	[66]
Nitrogen-doped CNTs encapsulated with Ni–Co alloy nanoparticles	445.6 at 700 °C 537.5 at 800 °C 801.4 at 900 °C	-	Methylene blue Methylene orange Phenol	-	[67]
Co_0·5_Ni_0·5_FeCrO_4_ spinel nanoparticles decorated with UiO-66-based metal–organic frameworks grafted onto GO and oxidized SWCNT	-	-	4-Nitrophenol	-	[106]
Ag-Fe_3_O_4_-CNT composite	375	-	O-nitro phenol P-nitro phenol 2-Methyl-p-nitrophenol Methylene blue	-	[107]
Iron manganese oxide-modified multiwalled CNT	211.3	-	Basic violet Acid orange	165.29 403.23	[72]
Zn@Cu–Fe_2_O_4_–NC–CNT	-	36.14	Azure-II	50.25	[73]
CNT/MgO/CuFe_2_O_4_ magnetic composite powder	127.58	12.137	Methyl violet Nile blue	36.46 35.60	[74]
Co_0.5_Ni_0.5_Fe_2_O_4_ NPs grafted onto CNTs	142.93	-	Methylene blue Methyl orange Congo red Rhodamine B	88.05 48.60 291.3 120.78	[32]
Pd–Fe dual-metal nanoparticles anchored in an interface of double-layered carbon nanotubes/nitrogen-doped carbon	163	-	4-Nitrophenol	-	[108]
Graphene oxide/multiwalled carbon nanotube/Fe_3_O_4_/SiO_2_	79.7,	-	Paracetamol Caffeine	-	[92]
Magnetite/multiwalled carbon nanotubes/metal–organic framework composite	-	21	Esters Dimethyl phthalate Diethyl phthalate Diallyl phthalate Methylparaben Butylparaben	-	[104]
CNT–FeNi_3_/DFNS/Cu(II) magnetic nanocomposite	341	19.7	Tetracycline	-	[86]
Fe–Cu-doped multiwalled carbon nanotubes	237.8–323.5	-	Paracetamol	-	[93]
Nitrogen-doped carbon nanotubes encapsulating Fe/Zn nanoparticles	-	-	Sulfamethoxazole	-	[90]
CNT-COOH/MnO_2_/Fe_3_O_4_ nanocomposite	114.2	0.46	Paracetamol Ibuprofen	80.645 103.093	[94]
Co_0.5_Mn_0.5_Fe_2_O_4_-CNT	108.20	0.61	Pentachlorophenol	43.2	[33]
Co_0.5_Ni_0.5_Fe_2_O_4_-CNT	95.52	0.61	Pentachlorophenol	40.8	[33]
Co_0.5_Cu_0.5_Fe_2_O_4_-CNT	112.04	0.42	Pentachlorophenol	35.1	[33]
Co_0.5_Zn_0.5_Fe_2_O_4_-CNT	96.05	0.40	Pentachlorophenol	33.9	[33]
Nitrogen-doped carbon nanotubes modified with magnetic Co_0.5_Cu_0.5_Fe_2_O_4_ nanoparticles	85.04–95.64	0.415	Chlorophenol	-	[109]
Bi_2_O_2_CO_3_/CNT/ZnFe_2_O_4_	-	25	2,4-Dimethyl phenol	-	[110]
Fe-doped graphitic carbon nitride coupled Ag_3_VO_4_ compounded with CNTs	-	-	2,4-Dimethyl phenol	-	[111]
FeOx/MnOy-modified oxidized CNTs	133–140	-	Rhodamine B	-	[71]
CNTs/β-cyclodextrin/MnFe_2_O_4_	166	25.706	Tetracycline	40.36	[87]

### 3.2. Polymer-Based Composites

Polymer-based magnetic composites are generally defined as organic polymer matrices embedded with inorganic magnetic components, mainly Fe_3_O_4_, Fe_2_O_3_/γFe_2_O_3_, CoFe_2_O_4_, ZnFe_2_O_4_, and NiFe_2_O_4_ [22,27]. In addition, polymers can be used as coating layers for magnetic nanoparticles to form chemically or physically anchored core–shell structures. The polymeric covers act as protective layers while also providing active sites for pollutant adsorption, and thus they are valuable materials for improving water decontamination performance [15].

What makes polymers interesting for the formation of various composites is their low weight, easy processing, and inexpensive fabrication [22]. Polymer-functionalized nanocomposites also benefit from more advantageous physicochemical characteristics compared to each of the system components, such as enhanced surface area-to-volume ratio, higher interfacial reactivity, and augmented mechanical properties. In addition, polymers endow composites with a highly tunable adsorption behavior, which makes them appealing for water treatment and purification technologies [27]. Moreover, in combination with magnetic elements, polymer-based composites offer enhanced nanoparticle stability, helping them avoid processes like oxidation and flocculation [22]. Furthermore, the magnetic components enable stable material recovery, providing easy separation from treated water and recycling performance to the polymer-based adsorbents [49,141,142].

With these advantages in mind, several research studies have investigated the potential of ferrite-supported nanocomposite polymers for the removal of different contaminants from aqueous samples [143]. Specifically, various ferrites have been combined with polymers like chitosan [144,145,146,147,148], polypyrrole [149], polyaniline [150], polyimide [151], and polyvinyl alcohol [152] to offer effective solutions for the removal of organic [144,145,146,147,150,151,152,153,154,155,156] and inorganic [148,149] pollutants (Table 4).

### 3.3. Hydrogel-Based Composites

Hydrogels can be considered a special class of polymeric materials due to their unique network structure and additional advantageous properties that render them suitable for a broad range of applications [157]. Hydrogels present a three-dimensional porous network of hydrophilic polymer chains that create an ideal adsorption and storage medium for large amounts of water, thereby being an appealing option for aqueous pollution remediation. In more detail, water permeates the hydrogel through capillary effect and osmolarity, which are mechanisms correlated with the hydrophilic functional groups, such as hydroxyl, carbonyl, carboxyl, and amino groups [157,158].

Furthermore, hydrogels made of biopolymers exhibit distinct beneficial characteristics, including safety, environmentally friendly nature, easy handling, tunable dimensions, and diverse morphology [158,159]. Moreover, hydrogels are excellent matrixes for the incorporation of different fillers, leading to synergistically acting composites. Embedding magnetic structures into hydrogels has been explored as a particularly effective option for water remediation applications, enhancing the mechanical properties of the composite material, augmenting its stability, and improving the electrical and thermal properties of hydrogels. Moreover, the addition of metallic magnetic particles endows the material with catalytic activity for degrading captured pollutants and provides the possibilities of remotely controlled swelling and the adsorption/desorption of analytes and collection from wastewater systems by adjusting the external magnetic field [157,158,159]. Besides, the endowed magnetism facilitates the separation of hydrogel composite beads after contaminant extraction. Furthermore, the used adsorbents can be conveniently regenerated and recycled through successive adsorption and washing rounds, considerably diminishing the economic costs of water treatment in practical applications [159,160].

Hydrogels have been especially recognized for the adsorptive removal of dyes from contaminated water for nearly two decades since various hydrogel-based composites started being developed [159]. According to the literature, alginate–chitosan hydrogels can reach dye removal capacities of larger than 100 mg/g (sometimes even surpassing 2000 mg/g), while metal absorption capacity can range between 38 mg/g and more than 440 mg/g [161]. Recent studies in the field (Table 5) have uncovered the potential of hydrogel-based magnetic composites for the removal of various organic dyes (e.g., reactive orange 16 [162], methylene blue [163,164,165,166,167,168], rhodamine B [166], methyl orange [168], and malachite green [168]) and environmentally harmful inorganic contaminants (e.g., Cd^2+^ [169], Pb^2+^ [168,170,171], Hg^2+^ [168], Ni^2+^ [168], Mn^2+^ [171], Cu^2+^ [171], Al, K, Se, Na, V, and S [158]).

### 3.4. Metal–Organic Framework (MOF)- and Covalent Organic Framework (COF)-Based Composites

Metal–organic frameworks (MOFs) and covalent organic frameworks (COFs) represent special classes of polymeric materials that have made great progress in recent years. MOFs are porous coordination polymers that can self-assemble from organic ligands and metal ions or clusters of metal ions to create various geometries (e.g., pyramidal, trigonal bipyramidal, square, octahedral, and tetrahedral). Numerous metal ions have been considered for MOF development, including Cu^2+^, Mg^2+^, Cd^2+^, Zn^2+^, Co^2+^, Ca^2+^, Fe^3+^, Al^3+^, Ti^3+^, Ln^3+^, and Zr^4+^, while organic components are generally amines, carboxylates, sulfonates and phosphates [27,172].

**Figure 5:**
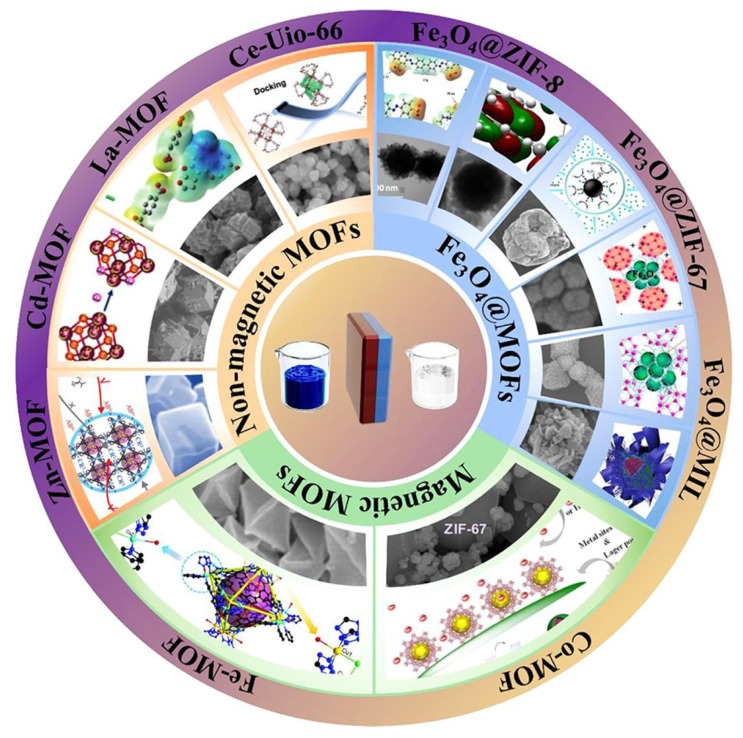
Overview of various MOFs. Reprinted from an open access source [173].

Many MOFs can be formed by different combinations between the aforementioned materials (Figure 5), yet some of them have gained more relevance. For instance, the MIL (Materials of Institut Lavoisier) series of MOFs have been produced from transition metals or metal ions (or clusters) from the lanthanide series and linkers of terephthalic acid or trimesic acid. ZIFs (zeolitic imidazolate frameworks) are another important series of MOFs that are produced through the coordination of metal ions and imidazole ligands. Other interesting MOFs are the UiO (University of Oslo) series, based on zirconium, and Cu-BTC or HKUST, based on copper [172].

Given their versatility, unique structure, and appealing physicochemical properties (e.g., high chemical stability, presence of active metal sites, large surface area, and tunable pore size), MOFs have attracted interest for wastewater treatment [27]. Among the many possible structural compositions, magnetic MOFs can provide particularly promising results, as they can allow for easy and high-efficient recycling [173]. By applying an external magnetic field, the MOF-based adsorbent used can be easily separated from water samples, further regenerated by washing with common solvents (e.g., ethanol), and reused several times without significant loss in its adsorption capacity [27].

The main magnetic component associated with MOFs is iron oxide, due to its superparamagnetism, biocompatibility, and desirable stability against chemicals. Thus, various iron oxide MOFs started to be employed in environmental applications to remove a wide range of organic and inorganic pollutants from contaminated water samples [173].

Magnetic MOFs have been rendered especially valuable for the adsorption of dyes, which has been demonstrated to be effective in numerous recent studies. They have been successfully used for decontaminating water from organic dyes like methylene blue [174,175,176,177,178], rhodamine B [174], methyl orange [179,180,181], indigo carmine [175], Congo red [182], AB92 [183], and DR31 [183]. Moreover, studies have been performed on other organic contaminants as well, with investigations being reported for the removal of an important number of drugs, including but not limited to ciprofloxacin [180,184], norfloxacin [180,184], tetracycline and its derivatives [34,185,186,187], diclofenac sodium [186,188], and ofloxacin [189]. For clarity, an at-glance perspective on MOF-based magnetic composites for organic pollutant removal is presented in Table 6.

In addition, recent articles have also reported on the potential of magnetic MOFs for the adsorption of heavy metal ions, as summarized in Table 7. Studies have shown that magnetic MOFs can be used for the efficient removal of a series of harmful metals from contaminated water samples, such as Co(II) [190], Cr(VI) [191,192,193,194,195], As(V) [196], Hg(II) [197,198], Pb(II) [187,192,199,200], U(VI) [201], Cu(II) [199,202,203], and Cd(II) [204].

Similar to MOFs, COFs have drawn attention to the adsorptive removal of targeted environmental pollutants [172]. Constructively, COFs are ordered crystalline porous polymers containing light elements connected to organic monomers through robust covalent bonds with ordered π structure [15,172,205]. Generally, COFs are MOF derivatives, displaying comparable surface areas; low densities; and well-defined pore size, topology, and framework. Other advantageous properties include their ease of functionalization, thermal and chemical stability, and ordered channel architecture [15,205].

Nonetheless, in most situations, COFs made in powder form have the disadvantages of lengthy operation, a significant agglomeration tendency, and low recyclability, which severely limits their environmental applications. Combining COFs with magnetic components emerged as an interesting solution to address these difficulties. Magnetic COF-based composites have an excellent adsorption capacity due to their well-developed pore structure, and they are endowed with superior magnetic responsiveness that facilitates their separation, recovery, and recycling. Owing to their unique qualities, magnetic COFs hold great promise for water remediation [205], and their potential for the elimination of toxic contaminants has been reported in a series of recent studies (Table 8). Magnetic COF-based composites have proved effective for the removal of various organic (e.g., triclosan [206], triclocarban [206], polycyclic aromatic hydrocarbons [207], bisphenols [208,209], methyl orange [210], diclofenac [211], and sulfamethazine [211]) and inorganic (e.g., Cr(VI) [209,212], Pb(II) [213], Hg(II) [214,215], Au(III) [216], and UO_2_^2+^ [217]) pollutants.

**Table 6:** Magnetic metal–organic framework-based composite adsorbents for organic pollutant removal from contaminated water.

Adsorbent	Surface Area (m^2^ g^−1^)	Magnetic Saturation (emu g^−1^)	Pollutant	Adsorption Capacity (mg g^−1^)	References
Polyoxometalate/CoFe_2_O_4_/metal–organic framework magnetic core–shell nanocomposites	799.56	13.7	Rhodamine B Methylene blue	153.84 200	[174]
Composite based on metal–organic frameworks and Fe_3_O_4_ nanoparticles	-	30.1	Anthracene	12.7	[218]
Composite material graphene oxide/MIL-101(Fe)	888.29	-	Methyl orange	186.20	[179]
Ag NPs supported on the magnetic Al-MOF/PDA	54.31	26.62	Ciprofloxacin Norfloxacin Methyl orange	-	[180]
Ce-MOF@Fe_3_O_4_@activated carbon composite	-	21.39	Methylene blue Indigo carmine	84.9 85.5	[175]
Magnetic nanocomposite based on Zn/Fe-MIL-88B	186–216	-	Chlortetracycline	11.7–359.2	[185]
Magnetic Fe_3_O_4_-PSS@ZIF-67 composites with core–shell structure	1041.90	-	Methyl orange	738	[181]
Yolk-shell Fe_3_O_4_@MOF-5 nanocomposites	203	46.57	Methylene blue	-	[178]
La-MOF-NH_2_@Fe_3_O_4_	36.1	15.54	Congo Red	716.2	[182]
Superparamagnetic MOF@GO Co-based hybrid nanocomposite	71.47	56.4	Methylene blue	67	[176]
Superparamagnetic MOF@GO Ni-based hybrid nanocomposite	-	47.0	Methylene blue	54	[176]
Fe_3_O_4_ NPs incorporated into the zeolitic imidazolate framework lattice (Fe_3_O_4_@ZIF-8)	1206	37.87	Methylene blue	-	[177]
PPI–Dendrimer-Functionalized Magnetic MOF (Fe_3_O_4_@UiO-66@PPI)	120	10.5	AB92 DR31	122.5 173.7	[183]
Magnetically functionalized Zr-MOF (Fe_3_O_4_@MOF-525)	427	7.48	Tetracycline Diclofenac sodium	277 745	[186]
β-cyclodextrin-modified Fe_3_O_4_@MIL-100(Fe) composite	2.60	9.40	Fungicides	64.52–102.10	[219]
Composites with a magnetic Fe_3_O_4_ core and a MIL-101 (Cr) MOF shell	803	19.6	Polyaromatic hydrocarbons	-	[220]
Fe_3_O_4_/HKUST-1 magnetic copper-based MOFs	327.9	44	Ciprofloxacin Norfloxacin	538 513	[184]
Magnetic Fe_3_O_4_@ZIF-67 composites	-	60.9	Tetrabromobisphenol A	-	[221]
Hydrophobic carboxyl-functionalized ionic liquid encapsulated into Fe_3_O_4_@Zr-MOFs	685	48.8	Ofloxacin	438.5	[189]

**Table 7:** Magnetic metal–organic framework-based composite adsorbents for inorganic pollutant removal from contaminated water.

Adsorbent	Surface Area (m^2^ g^−1^)	Magnetic Saturation (emu g^−1^)	Pollutant	Adsorption Capacity (mg g^−1^)	References
Zr-based magnetic metal–organic framework composite (Fe_3_O_4_@SiO_2_@UiO-66-Glu)	633.5	17.40	Co(II)	178.6–270.3	[190]
Magnetic ZIF-67 MOF@aminated chitosan composite beads	220.76	10.90	Cr(VI)	119.05	[191]
Magnetic Fe_3_O_4_@UiO-66 composite	33.12	26.5	As(V)	73.2	[196]
Magnetic materials with functionalized titanium-based MOF composite (SNN-MIL-125(Ti)@Fe_3_O_4_)	195.82	13.06	Hg(II)	511.4	[197]
Magnetic Zr-MOF named Ni_0.6_Fe_2.4_O_4_-UiO-66-PEI	22	4.11	Pb(II) Cr(VI)	273.2 428.6	[192]
Magnetic MOFs/graphene oxide (Fe_3_O_4_@HKUST-1/GO)	72.23	-	U(VI)	202.84–268.82	[201]
Surfactant-functionalized magnetic MOF@MOF adsorbent (Fe_3_O_4_@UiO-66@UiO-67/CTAB)	115.94	36.05	Cr(VI)	932.1	[193]
Fe_3_O_4_@metal–organic framework@covalent organic framework (Fe_3_O_4_@MOF@COF)	-	16	Cu(II)	37.29	[202]
Citrate capped Fe_3_O_4_@UiO-66-NH_2_ MOF	572.13	3.07	Cr(VI)	743	[194]
Fe_3_O_4_@ZIF-8 core–shell magnetic composite	724.7	37.26	Pb(II) Cu(II)	714.7 299.7	[199]
Magnet-responsive Fe_3_O_4_@ZIF-8	896	27	Cu(II)	345	[203]
Magnetic Zr-MOF@polypyrrole (Fe_3_O_4_@UiO-66@Ppy)	52.49	19.75	Cr(VI)	259.1	[195]
Multifunctional composite Fe_3_O_4_/MOF/L-cysteine	413.67	-	Cd(II)	248.24	[204]
Fe_3_O_4_@DTIM-MOF@SH composite	827	13	Hg(II)	756.9	[198]
Fe_3_O_4_@ZIF-8 composite	1722	13.4	Pb(II)	276.06	[187]
Polyacrylic acid capped Fe_3_O_4_–Cu-MOF	332.07	-	Pb(II)	610	[200]
Non-core–shell Fe_3_O_4_@ZIF-67 composites	-	-	Phosphate	116.59	[222]

**Table 8:** Magnetic covalent organic framework-based composite adsorbents for various pollutants’ removal from contaminated water.

Adsorbent	Surface Area (m^2^ g^−1^)	Magnetic Saturation (emu g^−1^)	Pollutant	Adsorption Capacity (mg g^−1^)	References
Core–shell structured magnetic covalent organic framework nanocomposites	55.71	48.4	Triclosan Triclocarban	-	[206]
Bouquet-shaped magnetic porous nanocomposite made of TpPa-1 grafted on surface-modified Fe_3_O_4_ nanoparticles	247.8	40.1	Polycyclic aromatic hydrocarbons	-	[207]
Porous nanospheres with a magnetic core and a tunable TpBD shell	272.6	22	Bisphenol A Bisphenol AF	160.6 236.7	[208]
Magnetic porous covalent triazine-based framework composites	930–1149	1.1–5.9	Methyl orange	291	[210]
Magnetic covalent organic framework (TpPa-1) with β-ketoenamine linkage	485.2	19.5	Bisphenol A	1220.97	[209]
Fe_3_O_4_ particles grown in the pore channels of COFs	2245	5.2	Diclofenac Sulfamethazine	40.4 55.24	[211]
Fe^0^ nanoparticles immobilized on porous TpPa-1 covalent organic framework	102.97	-	Cr(VI)	516	[212]
Bimetal oxide MnFe_2_O_4_ incorporated onto β-ketoenamine linked TpPa-1	152.5–450.5	11.52	UO_2_^2+^	1235.01	[217]
Magnetic covalent organic framework (TpPa-1) with β-ketoenamine linkage	485.2	19.5	Cr (VI)	245.45	[209]
Magnetic organic framework adsorbent (Ni_0.6_Fe_2.4_O_4_-HT-COF)	-	39.83	Pb(II)	411.80	[213]
Thiol-functionalized magnetic covalent organic frameworks	181.5	19.6	Hg(II)	383	[214]
Fe_3_O_4_ decorated porous melamine-based covalent organic framework	344–600	0.75–3.59	Hg(II)	97.65	[215]
Magnetic *β*-ketoenamine COF (MTpPa-1)	538.60	6.59	Au(III)	1737	[216]

### 3.5. Silica-Based Composites

Silica is another material recognized and exploited for its three-dimensional network structure. It consists of SiO_4_ ending with oxygen atoms connected via siloxane or silanol groups. Silanol groups are particularly relevant for water decontamination applications, as they provide beneficial surface chemistry for the adsorption of molecules and metal cations through complex formation (an interaction that can be enhanced by pH modification). Moreover, silica exhibits easy grafting of additional functionalities, including photocatalyst grafting for dye degradation. Hence, plain silica can be successfully employed in removing various pollutants, including aromatic compounds, organic dyes, and heavy metals [15,223].

Nonetheless, even better outcomes can be obtained when combining silica with other materials to create magnetic composites that can be easily removed from wastewater and further reused. From a constructive point of view, similar possibilities to magnetic polymer-based composites are often involved: core–shell structures and dispersed magnetic particles in a silica matrix. Using nonporous or mesoporous silica to cover iron oxide nano-/microparticles is an appealing method for protecting the magnetic core from leaching and oxidation while reducing particle aggregation tendency [15]. Moreover, the application of a solid silica coating on iron oxide particles improves their stability and restricts their dissolution, as silica ring molecules block the diffusion of structures larger than oxygen. Physical damage to the silica layer is the sole method through which the chemical resistance of magnetite nanoparticles coated with solid silica can be diminished [224]. In addition, the augmentation of silica-based composites with magnetic materials enables facile separation from aqueous solutions through the application of external magnetic forces, thus simplifying the adsorption process and enhancing the overall adsorption capacity. After contaminant removal from the treated water, the magnetic adsorbent can be easily regenerated (e.g., acid treatment) and reused for successive decontamination procedures [225].

Given these advantageous properties, it is no surprise that silica-based magnetic composites have been explored as unconventional effective adsorbents, with numerous studies being focused on dye removal from contaminated waters [223,226] (Table 9). Organic compounds (e.g., phenanthrene [227], methylene blue [228,229,230,231,232], Congo red [230], glyphosate [233], methylene red [234], doxycycline [235], acid blue 25 [236], fenpropathrin [237], cyhalothrin [237], S-fenvalerate [237], bifenthrin [237], bisphenol A [238], and methyl orange [239]) have been successfully removed from water in recent studies involving different combinations between silica, magnetic particles, and other materials.

Researchers have recently developed modified magnetic mesoporous materials capable of eliminating heavy metals via charge transfer and electrostatic attraction mechanisms [226]. For instance, EDTA-modified magnetic mesoporous microspheres have been considered for Cr^3+^ removal [240], while iron oxide magnetic nanoparticles coated with silica have been employed in Pb^2+^ adsorption [241].

### 3.6. Aerogel-Based Composites

Aerogels can be considered a distinctive category of materials with porous structures that can be accomplished from different raw components. Aerogels have a very low density, consisting of 90–99% air. Their unique 3D network of interconnected pores is generally developed by crosslinking polymeric nanoparticles, removing the solvent from the obtained gel, and then filling the pores with air [27,242,243]. Nonetheless, numerous other base materials can be employed for fabricating aerogels due to the progress made in the preparation and drying processes. Currently, these materials include organic aerogels (made from chitosan, gelatin, cellulose, etc.), inorganic aerogels (made from silica, titania, alumina, etc.), carbon aerogels (made from graphene or carbon nanotubes), and others, reflecting the uniqueness, versatility, and potential of aerogel-based materials [242,243].

Silica-, polymer-, and carbon-based aerogels have entered the market in several fields, including transportation, construction, and coatings [242], with interesting prospects also noted for catalysis, adsorption, and biomedicine [243]. These lightweight materials have also attracted renewed interest in water decontamination [27]. Their high surface area, tunability in terms of hydrophobicity/hydrophilicity, and readily recyclability reinforce their potential for water treatment alternatives. In addition, aerogels benefit from their nontoxic characteristic, nonflammability, and easily disposable nature [242,244].

Besides the useful properties of monocomponent aerogels, functionalizing these porous materials with synergistic compounds unveils new avenues for their high-scale utilization. In particular, converting pristine aerogels into magnetic composites has contributed to extending the performance of these materials and endowed them with superparamagnetic properties. Magnetic aerogels can maintain their magnetic properties and adsorption ability throughout several decontamination cycles, as they can be easily recollected from water samples, regenerated, and reused in further treatments [244].

For matrix material, the most employed aerogels are silica-based composites, followed by aerogels based on carbon, natural polymers/cellulose, metals, synthetic polymers, alumina, and clays [244]. Regarding decontamination potential, various recent studies (Table 10) have highlighted the use of magnetic aerogel-based composites to remove both organic and inorganic pollutants. Encouraging results have been obtained for the elimination of organic contaminants, like methylene blue [245,246,247,248,249], acid orange [245], Congo red [246,249,250], crystal violet [246], methyl orange [246,248], malachite green [251,252], reactive black 5 [253], and organic solvents and oils [254]. Few interesting studies have also reported on the possibility of adsorbing heavy metals ions from contaminated water, including Cr(III) [249], Cr(VI) [245], As(V) [245], Cd(II) [249,255], Cu(II) [249], and Pb(II) [249].

### 3.7. Biochar-Based Composites

Biochar represents a stable substrate obtained from biomass through the combustion of organic materials under low or no oxygen conditions [257]. Various materials can be turned into biochar, with much focus placed on the exploitation of waste products [258]. Biochar-based materials have heterogeneous properties, and their characteristics depend on the raw materials utilized and production conditions [257,258]. Common feedstocks are switchgrass, hardwoods, peanut hulls, corn hulls, pecan shells, bark, rice, sugarcane, leaves, paper sludge, cow manure, poultry manure and litter, sewage sludge, and aquaculture waste. Biochar can assist in decreasing people’s aversion to discarding stream items by reducing both dampness and odor through the process of pyrolysis [257].

Biochar-based materials have numerous attractive physicochemical features, including high surface area, stable structure, microporosity, high carbon content, cation exchange capacity, and charged surface functional groups [257,258]. These properties raised interest in biochar for use in environmental applications, as they enable the immobilization or removal of contaminants from soil, water, and air [259].

Although biochar can be used to absorb organic pollutants such as pesticides and herbicides from contaminated water, it also hinders bacteria’s ability to decompose these substances, extending their environmental persistence. Metals can also be chemically or physically adsorbed onto biochar-based materials, unlike organic compounds. Biochar does not impede the microbial degradation of inorganic contaminants [257]. To further improve their adsorbent potential in decontamination applications, biochars can be tailored through various chemical and physical modification methods, the incorporation of different materials, and magnetic functionalization [260].

Recent studies have particularly investigated the incorporation of metal ions (e.g., magnesium, silver, zinc, and copper) onto the surface and within the pores of biochar. The resulting materials exhibited a considerably enhanced adsorption capacity compared to pristine biochar due to the presence of two solid phases (i.e., metal oxide nanocrystals and biochar matrix) that contribute to contaminant removal ability through mechanisms like hydrogen bonding, precipitation, electrostatic precipitation, and ligand exchange [259].

When the metal oxides introduced in the porous carbon platform possess magnetic properties, the obtained composite may display permanent magnetism after pyrolysis, leading to improved decontamination efficiency [260]. Moreover, magnetic biochar-based adsorbents can be easily separated from water samples with the aid of a permanent magnet. After recovery, contaminants like heavy metal ions can be desorbed from the composite through treatment with a strong base, and the adsorbent can be further regenerated through HCl treatment [261].

Unlike unmodified biochar, magnetic biochar-based composites offer better outcomes in the removal of water pollutants, including heavy metals, dyes, drugs, and pesticides [262]. For clarity, Table 11 summarizes recently reported magnetic biochar-based adsorbents that have shown good promise for eliminating various organic [263,264,265,266,267,268,269,270] and inorganic [271,272,273,274,275,276,277,278] contaminants.

### 3.8. Clay-Based Composites

Another upcoming direction for water and wastewater treatment consists of developing clay-based composite materials. Clays are naturally occurring absorbents found abundant in sedimentary rocks in the form of hydrated phyllosilicates. The base unit of these materials is SiO_4_^4−^. Three of each tetrahedron’s apical oxygen atoms are shared with another tetrahedron, placing the fourth apical oxygen vertically on the sheet [15,27,279,280].

Clay minerals have the advantages of being cheap and widely available, features that allow them to be explored and exploited for environmental applications. They also benefit from high porosity, high surface area, hydrophilic character, and natural net negative charge on their configuration. Additionally, clays have great adsorption capability, swelling capacity, and ability to interleave and/or graft different moieties. These properties make clays valuable materials for water decontamination purposes, with a special focus on the removal of cationic particles and heavy metals from aqueous solutions [15,279].

Different types of nanoclays can be involved in water treatment, including kaolin, bentonite, montmorillonite, illite, micas, and kaolinite [279]. Moreover, these materials can be further modified by incorporating magnetic components to optimize the systems toward more proficient and economical adsorption platforms [27]. The obtained magnetic clay-based composites exhibit improved physicochemical characteristics, such as high electrical and chemical resistance, strong mechanical properties, superparamagnetism, saturation magnetization, and enhanced specific surface area [279]. These magnetic adsorbents also allow for easy separation via an external magnetic field without affecting water turbidity, and their stability and reusability make them versatile materials for removing cationic and anionic pollutants individually or concurrently [281].

Furthermore, other components can be added to the composite (e.g., surfactants, polymers, and other substances with functional groups of interest) to attract the adsorbate more effectively [280]. With their additional properties, magnetic clay-based composite materials can be used to eliminate diverse pollutants, including heavy metals, dyes, drugs, and other organic contaminants, through various processes (e.g., adsorption, chemical treatment, oxidation, and photo-oxidation) [279,280].

In more detail, recent studies (Table 12) have demonstrated the capacity of magnetic clay-based composites to remove organic (e.g., direct red 23 [282], crystal violet [283], acid red [283], Congo red [284], methylene blue [285,286,287], sunset yellow [288], Nile blue [288], naphthol blue-black [289], enrofloxacin [290], tetracycline [291], ciprofloxacin [291], phenol [292], p-nitrophenol [292], p-cresol [292], atrazine [293], and bisphenol A [294]) and inorganic (Cr(VI) [284], Cu(II) [284,295,296], Pb(II) [284,295], Cd(II) [285], Ni(II) [295], F [297], and Sr(II) [298]) pollutants from water and wastewater.

### 3.9. Summative Discussion and Remaining Challenges

In recent years, extensive interest has been directed toward developing carbon- and polymer-based composite materials combined with magnetic particles to act upon numerous organic and inorganic contaminants, which may pose significant environmental problems. These composites provide facile separation from tested solutions; are chemically stable; and have good recyclability, enhanced porosity, high surface area, and great adsorption capacity, properties well found on the list of requirements for an ideal adsorbent.

However, several limitations remain and must be addressed before coming onto the market with better-performing water remediation solutions.

Magnetic composites are widely used in wastewater treatment, especially due to their high flocculating and ferromagnetic properties [49]. However, depending on the material they are associated with (Table 13), the magnetism of metallic particles may be shielded by the covering layers, leading to overall poorer magnetic properties. Hence, special attention must be given when choosing the materials to optimize targeted properties while preserving enough magnetism to enable efficient separation.

High variability can be observed between the adsorption capacity of the reviewed magnetic adsorbents, a property that depends not only on the matrix material but also on the target contaminants. Considering the tabulated composites for which data were available, the variation in the adsorption capacity could range between several orders of magnitude for each base material. For instance, for CNT-based magnetic composites, an adsorption capacity of 0.95 mg g^−1^ was registered for dimethyl phthalate in the case of magnetic multitemplate molecularly imprinted polymer@MWCNTs [112], while a more complex-structured adsorbent (i.e., core–shell ZIF-67/ZIF-8-derived sea urchin-like cobalt/nitrogen Co-doped CNT hollow framework) allowed for the adsorption of 8862.5 mg g^−1^ for methylene blue [59]. For graphene-based magnetic composites, the highest reported adsorption capacity of 1590 mg g^−1^ was observed for tetracycline adsorbed by magnetic graphene oxide/ZnO nanocomposites [120], whereas a maximum of 3 mg g^−1^ was reported for chromium ions removed by magnetic graphene oxide [133]. However, this does not necessarily imply that graphene-based magnetic composites are bad adsorbents for inorganic contaminants, as other compositions could lead to better adsorption properties (e.g., Fe_3_O_4_/SiO_2_–graphene oxide had a 385 mg g^−1^ adsorption capacity for Pb(II) [128]). For polymer-based magnetic structures, the adsorption capacities ranged between 6.7 mg g^−1^ for fluoride ions captured by cobalt ferrite–chitosan magnetic composites [148] and 23 g g^−1^ for spill oils adsorbed by magnetic mesoporous lignin [156], indicating the exceptional potential of the latter material in water decontamination. For hydrogel-based composites, the lowest reported adsorption capacity value was 1.83 mg g^−1^ for Mn(II) (i.e., magnetic sodium alginate/carboxymethyl cellulose composite hydrogel [171]), while the highest was 1603 mg g^−1^ for methylene blue (i.e., polyacrylamide/chitosan/Fe_3_O_4_ composite hydrogels [163]). MOF-based composites ensured the highest adsorption capacity for Cr(VI) (i.e., 932 mg g^−1^ for Fe_3_O_4_@UiO-66@UiO-67/CTAB [193]), while the lowest was reported for anthracene (i.e., 12.7 mg g^−1^ composite based on MIL-101 and Fe_3_O_4_ [218]). In a similar fashion, the highest adsorption capacity reported for COF-based magnetic adsorbents was 1734 mg g^−1^ for metallic ions (i.e., Au(III) gold removal via magnetic β-ketoenamine COF (MTpPa-1) [216]) whereas the lowest was 40.4 mg g^−1^ for an organic compound (i.e., diclofenac removal via Fe_3_O_4_ particles grown in the pore channels of COFs [211]). In the case of silica-based magnetic composites, the available data for adsorption capacity ranged between ~2.45 mg g^−1^ for pyrethroid pesticides and 909 mg g^−1^ for acid blue 25, removed by mesoporous composite Fe_3_O_4_@SiO_2_@KIT-6 [237] and magnetic-SBA-15 crosslinked poly(acrylic acid) [236], respectively. Magnetic aerogels offer an exceptional adsorption capacity in general, regardless of contaminant type, with the numerical values starting from 83.5 mg g^−1^ for methyl orange (i.e., Fe_3_O_4_@PDA/CMC aerogel [246]) and reaching up to 537 g g^−1^ for organic solvents and oils (i.e., magnetic carbon nanosphere/graphene composite aerogels [256]). Biochar-based magnetic composites exhibited adsorption capacities in the range between 29.4 mg g^−1^ and 680 mg g^−1^ for tetracycline removal by magnetic Fe_3_O_4_ biochar [268] and Pb(II) removal by halloysite and coconut shell biochar magnetic composites [273], respectively. For clay-based materials, the lowest reported adsorption capacity value was ~9 mg g^−1^ for Cd(II) (i.e., magneto-carbon black-clay composite [285]), whereas the highest adsorption capacity was 368 mg g^−1^ for Pb(II) (i.e., EDTA-modified magnetic attapulgite chitosan gel beads [295]).

In addition, certain specific limitations have been encountered for each material. For instance, composites developed by combining carbon-based materials and magnetic particles were noted to improve the adsorption properties compared to the pristine carbon material, due to more available adsorption sites and enhanced porosity [15]. Nonetheless, graphene is still quite expensive and is not a feasible alternative for large-scale experimentation [22]. Certain improvements must also be made to reduce the cost of aerogel production to ensure their entrance into the market availability [242]. Similarly, despite promising laboratory-scale results, magnetic COF-based materials are difficult to scale up for industrial production, as they require complex and costly synthesis processes. Moreover, the introduction of magnetic nanoparticles in the structure of COFs may affect their crystalline structure, further impacting the specific surface area and adsorption capacity [205].

Several limitations have also been observed for the use of silica-based composites. Specifically, a proper assessment of the effects of the large-scale utilization of these materials and the implied environmental risks depending on other compounds introduced in their structure is lacking [226]. Similar considerations are warranted for all the tabulated magnetic composites, given that the materials were only tested in small-scale studies [15,279].

Another important aspect to be considered is the regeneration of developed unconventional adsorbents so that they do not become waste materials and lead to secondary pollution [22,205,280]. In this respect, after capturing the targeted contaminants and being separated from an aqueous solution, carbon- and polymer-based magnetic composites can be subjected to processes like thermal regeneration, ultrasonic treatment, chemical treatment, gamma irradiation, and microwave irradiation, ensuring their suitability for another decontamination cycle [22].

Moreover, most of the reviewed studies have been carried out on synthetic wastewater solutions, leaving a gap for how they would function in real-life applications, where samples generally present a mixture of pollutants. In addition to its more complicated composition, industrial water exhibits a wide pH range, significant water quality fluctuation, and variable chemical and biological stability [27,279,280,299].

## 4. Conclusions and Future Perspectives

In summary, magnetic composites hold great promise for water decontamination applications, increasing research interest in developing newer and better water remediation systems. The versatility, tunability, surface properties, and ease of separation of different magnetic nanoparticles have led to increased attention directed toward their inclusion in various composite materials. Numerous magnetic-based composites have been fabricated and tested to remove various organic and inorganic contaminants from aqueous solutions, offering encouraging prospects for replacing conventional water treatment methods.

Magnetic nano- and micromaterials based on iron, cobalt, nickel, magnetite, and maghemite have been explored in association with a wide range of other materials, either as matrices or as coatings for metallic cores. The most studied magnetic composites involved carbon- and polymer-based structures. Specifically, an impressive number of papers have reported the potential of carbon nanotube- and graphene oxide-based magnetic composites. Concerning polymers, there is broad material variability, with recent studies pointing to the potential of conventional natural and synthetic polymers, hydrogels, aerogels, MOFs, and COFs. Moreover, several important advancements have been reported in the use of silica-, biochar-, and clay-based magnetic composite materials, offering extensive possibilities for removing dyes, drugs, pesticides, heavy metal ions, and other contaminants from polluted water samples.

Despite the significant progress in the field, there is a need to bridge the gap between laboratory performance and real-world effects, necessitating the conversion of reported achievements to low-cost scalable technology. Thus, future studies should focus on composite materials that have shown the most promising results for synthetic samples and test them on real wastewater in a broader context to ensure their advancement to higher technological maturity levels. Moreover, extensive tests should also be performed on the environmental impact of utilizing recently developed materials (i.e., the pollution resulting from their production, implementation, and destruction/recycling). Minimizing environmental impact should be a priority when deciding the materials and synthesis routes for the water remediation systems to avoid falling under the trap of replacing current contamination with secondary pollution. Moreover, the economic dimension must be considered. In this respect, the feasibility of the developed magnetic composites depends on the cost of raw materials, the equipment and energy requirements for production, the possibility for the successive use of fabricated adsorbents, and the capacity to remove multiple contaminants simultaneously.

To conclude, remarkable progress has been registered in developing a wide range of magnetic composite materials that, through thorough further evaluation and testing, can soon become performant alternatives to conventional decontamination methods.

## Figures and Tables

**Table 2:** Magnetic graphene oxide-based adsorbents for organic pollutant removal from contaminated water.

Adsorbent	Surface Area (m^2^ g^−1^)	Magnetic Saturation (emu g^−1^)	Pollutant	Adsorption Capacity (mg g^−1^)	References
Magnetic graphene oxide/ZnO nanocomposites	83.2839	11.18	Tetracycline	1590.28	[120]
Magnetite/reduced graphene oxide nanocomposite	213	18.269	Phenazopyridine	14.064	[121]
Amino-functionalized mesoporous silica-magnetic graphene oxide nanocomposites	-	39.37	Oxytetracycline	-	[122]
Graphene oxide/MIL-88A(Fe) membrane	-	-	Methylene blue Rhodamine B Methyl orange	-	[115]
Magnetic graphene oxide@MIL-101(Fe)	134.1	30.3	Diazinon and atrazine pesticides	-	[124]
Fe_3_O_4_@ZnO@graphene oxide nanocomposite	-	7.02	Methyl orange	-	[116]
Fe_3_O_4_@graphene oxide nanocomposite	-	45.788	Methyl orange	-	[116]
Montmorillonite/graphene oxide/CoFe_2_O_4_	194.94	46.34	Methyl violet	97.26	[117]
CuFe_2_O_4_@GO	32.3	-	Methylene blue	25.81	[118]
CoFe_2_O_4_@GO	52.1	-	Methylene blue	50.15	[118]
NiFe_2_O_4_@GO	76.7	-	Methylene blue	76.34	[118]
Cobalt ferrite-reduced graphene oxide	-	62	Methylene blue	4.3497	[65]
Magnetic chitosan nanocomposites modified with graphene oxide and polyethyleneimine	-	29.31	Congo red Amaranth	162.07 93.81	[119]
Mesoporous silica–magnetic graphene oxide nanocomposite	31.68	26.98	Sulfamethoxazole	15.46	[123]

**Table 3:** Magnetic carbon nanotube- and graphene oxide-based adsorbents for heavy metal removal from contaminated water.

Adsorbent	Surface Area (m^2^ g^−1^)	Magnetic Saturation (emu g^−1^)	Pollutant	Adsorption Capacity (mg g^−1^)	References
Multiwalled CNTs doped with magnetic iron oxide and deposited in crosslinked chitosan	70.90	-	Cr(III) Cr(VI)	66.25 449.30	[131]
Sulfur-coated magnetic multiwalled CNT	-	8.2	Hg(II)	62.11	[135]
Magnetic Fe_3_O_4_@C@TiO2–nanotube composites	37.02–50.33	2.1–3.9	Pb(II)	-	[126]
Magnetic CNT composites	-	35.8	Cu(II)	-	[61]
Magnetic graphene oxide	-	38	Pb(II) Cr(III) Cu(II) Zn(II) Ni(II)	200.00 24.330 62.893 63.694 51.020	[127]
Magnetic hollow-sphere nanocomposite, graphene oxide–gadolinium oxide	50.91	55	As	216.70	[136]
Magnetic chitosan/graphene oxide/MnO_2_	-	-	Cr(VI)	78.2	[132]
Magnetic chitosan/graphene oxide/Al_2_O_3_	-	-	Cr(VI)	77.8	[132]
Magnetic chitosan/graphene oxide/SiO_2_	-	-	Cr(VI)	75.9	[132]
Fe_3_O_4_/SiO_2_–graphene oxide composite	-	18.2	Cd(II) Pb(II)	128.2 385.1	[128]
Silica-coated magnetic graphene oxide	-	22.58	Cr(III) Co(II) Ni(II) Cu(II) Cd(II) Pb(II) Ag(I)	79.91 62.18 64.79 70.77 62.09 82.58 54.90	[129]
Magnetic graphene oxide	-	-	Cr(VI)	3.197	[133]
Graphene oxide functionalized chitosan–magnetite nanocomposite	-	-	Cu(II) Cr(VI)	111.11 142.85	[134]
MnFe_2_O_4_/GO nanocomposite	-	28.8	Pb(II)	90	[125]
Magnetic chitosan nanocomposites modified by graphene oxide and PEI	-	29.31	As Hg(II)	220.26 124.84	[119]
3-aminopropyltrimethoxysilane-functionalized magnetic sporopollenin-based silica-coated graphene oxide	-	30	Pb(II)	323.5	[130]
MnFe_2_O_4_/GO nanocomposite	-	28.8	Pb(II)	625	[125]

**Table 4:** Magnetic polymer-based adsorbents for various pollutants’ removal from contaminated water.

Adsorbent	Surface Area (m^2^ g^−1^)	Magnetic Saturation (emu g^−1^)	Pollutant	Adsorption Capacity (mg g^−1^)	References
ZnFe_2_O_4_/chitosan magnetic particles	57.4	9.75	Diclofenac	188	[144]
Magnetic geopolymer/Fe_3_O_4_ composite	53.40	2.50	Acid green 16	400	[153]
Magnetic zinc ferrite–chitosan biocomposite	5.187	-	Crystal violet Brilliant green	14.3 20.0	[145]
CoFe_2_O_4_–chitosan composite	2	8.4	Congo red Methyl orange	15.60 66.18	[146]
Polypyrrole-modified Fe_3_O_4_/SiO_2_ magnetic composite	-	8	Congo red	361.43	[149]
Bio-magnetic membrane capsules from PVA–alginate matrix	-	11.02	Malachite green	500	[152]
Magnetic nanocellulose from olive industry solid waste	-	21.4	Methylene blue	166.67	[154]
Magnetic amine-functionalized chitosan	-	17.5	Diclofenac sodium	469.48	[147]
Magnetic β-cyclodextrin porous polymer nanospheres	70.63	44.8	Methylene blue	305.8	[155]
Polyaniline-coated Fe_3_O_4_ nanoparticles	-	40.4	Polycyclic aromatic hydrocarbons	-	[150]
Magnetic polyimide@ Mg-Fe-layered double hydroxides core–shell composite	-	26.38	Tetracycline 2,4-Dichlorophenol Glyphosate	185.53 176.06 190.84	[151]
Magnetic mesoporous lignin from date palm pits	640	37.81	Spill oils	23.01 g g^−1^	[156]
Zinc ferrite–chitosan magnetic composite	3.833	-	F^−^	6.9	[148]
Nickel ferrite–chitosan magnetic composite	4.187	-	F^−^	8.3	[148]
Cobalt ferrite–chitosan magnetic composite	3.197	-	F^−^	6.7	[148]
Polypyrrole-modified Fe_3_O_4_/SiO_2_ magnetic composite	-	8	Cr(VI)	298.22	[149]

**Table 5:** Magnetic hydrogel-based adsorbents for various pollutants’ removal from contaminated water.

Adsorbent	Surface Area (m^2^ g^−1^)	Magnetic Saturation (emu g^−1^)	Pollutant	Adsorption Capacity (mg g^−1^)	References
Chitosan/graphite/polyvinyl alcohol magnetic hydrogel microspheres	-	7.2	Reactive orange 16	196.3	[162]
Polyacrylamide/chitosan/Fe_3_O_4_ composite hydrogels	-	-	Methylene blue	1603	[163]
Chitosan–graphene oxide hydrogels with embedded magnetic iron oxide nanoparticles	22.37–25.83	32.56	Methylene blue	36.2	[164]
Hydrogel beads based on the incorporation of nanosilver/diatomite into calcium alginate	0.31	-	Methylene blue	128.21	[165]
Graphene quantum-dot-decorated magnetic graphene oxide-filled polyvinyl alcohol hybrid hydrogel	-	20.55	Methylene blue Rhodamine B	46.79 44.89	[166]
Fe_3_O_4_-modified chitosan-based *co*-polymeric magnetic composite hydrogel	-	0.178	Methylene blue	-	[167]
Magnetic hydrogel microspheres of lignin derivate	-	-	Methylene blue Methyl orange Malachite green	43 39 155	[168]
Polyvinyl alcohol composite hydrogels containing magnetic nanoparticles	-	-	Cd(II)	42.6	[169]
Magnetic hydrogel microspheres of lignin derivate	-	-	Pb(II) Hg(II) Ni(II)	33 55 23	[168]
Magnetic chitosan/alginate/Fe_3_O_4_@SiO_2_ hydrogel composites	-	0.30–4.1	Pb(II)	>220	[170]
Magnetic sodium alginate/carboxymethyl cellulose composite hydrogel	-	3.2	Mn(II) Pb(II) Cu(II)	1.83 89.49 105.93	[171]
Alginate hydrogel reinforced with cellulose nanofibers decorated with magnetic nanoparticles	17.02	-	Al K Se Na V S	22 13.2 19 11.1 44.4 13.7	[158]

**Table 9:** Magnetic silica-based adsorbents for various pollutants’ removal from contaminated water.

Adsorbent	Surface Area (m^2^ g^−1^)	Magnetic Saturation (emu g^−1^)	Pollutant	Adsorption Capacity (mg g^−1^)	References
PVP-modified Fe_3_O_4_@SiO_2_ nanoparticles	60.82	30.89	Phenanthrene	18.84	[227]
Fe_3_O_4_@SiO_2_–VTEOS–DMDAAC	-	-	Methylene blue	109.89	[228]
Carboxylated ethylenediamine functionalized Fe_3_O_4_@SiO_2_ nanoparticles	-	58.7	Methylene blue	43.15	[229]
Fe_3_O_4_@SiO_2_@Zn–TDPAT	-	>20	Methylene blue Congo red	58.67 17.73	[230]
Fe_3_O_4_@SiO_2_@UiO-67	-	20.9	Glyphosate	256.54	[233]
Raspberry-like supraparticles made of very small silica nanoparticles and SPIONs	193	>25	Methylene blue	93	[231]
Fe_3_O_4_@SiO_2_@NH_2_	-	>40	Methylene red	81.39	[234]
Fe_3_O_4_@SiO_2_@mSiO_2_-CD	119	30.99	Doxycycline	78	[235]
Magnetic-SBA-15 crosslinked poly(acrylic acid)	159	2.68	Acid blue 25	909.09	[236]
Mesoporous composite Fe_3_O_4_@SiO_2_@KIT-6	579	42.8	Fenpropathrin Cyhalothrin S-fenvalerate Bifenthrin	2.47 2.47 2.43 2.45	[237]
Ag/Fe,N-TiO_2_/Fe_3_O_4_@SiO_2_	-	5.82	Bisphenol A	-	[238]
Hollow-structured Fe_2_O_3_/Au/SiO_2_ nanorods	58.23	-	Methyl orange	-	[239]
Yolk-porous-shell SiO_2_@void@Ag/TiO_2_ nanospheres	702.8	-	Methylene blue	-	[232]
EDTA-modified magnetic mesoporous microspheres	337.02	29.49	Cr(III)	-	[240]
Iron oxide magnetic nanoparticles with SiO_2_ shell	270–275	1.28–1.34	Pb(II)	14.9	[241]

**Table 10:** Magnetic aerogel-based adsorbents for various pollutants’ removal from contaminated water.

Adsorbent	Surface Area (m^2^ g^−1^)	Magnetic Saturation (emu g^−1^)	Pollutant	Adsorption Capacity (mg g^−1^)	References
Hexagonal boron nitride nanosheets (*h*-BNNSs) based on magnetic hybrid aerogels	104.6	74.6	Methylene blue Acid orange	415 286	[245]
N-doped magnetic carbon aerogel	94	85	Congo red	431	[250]
Multifunctional magnetic carboxymethyl chitosan (Fe_3_O_4_@PDA/CMC) aerogel	106.7	13.69	Methylene blue Crystal violet Methyl orange Congo red	217.43 262.27 83.47 92.83	[246]
Carboxymethylcellulose-based citric acid crosslinked magnetic aerogel	-	-	Methylene blue	83.6	[247]
NiCo-loaded reduced graphene oxide aerogel microspheres	253.9	43.8	Organic solvents and oils	107–270 g g^−1^	[254]
Magnetic carbon nanospheres/graphene composite aerogels	787.92	22.47	Organic solvents and oils	187–537 g g^−1^	[256]
Aerogels based on reduced GO decorated with nanoparticles of iron oxides Fe_3_O_4_ and γ-Fe_2_O_3_	670	-	Methylene blue Methyl orange	1501 1390	[248]
Fe-doped silica aerogel composite	240	-	Malachite green	1592	[251]
Amphiprotic cellulose-mediated graphene oxide magnetic aerogels	-	8.61	Congo red Methylene blue	282 346	[249]
Magnetic bacterial cellulose nanofiber/graphene oxide polymer aerogel	214.75	26.59	Malachite green	270.27	[252]
Polyaniline/hexaferrite aerogels supported by poly(vinyl alcohol)	-	7.7–12.5	Reactive black 5	-	[253]
Hexagonal boron nitride nanosheets (*h*-BNNSs) based on magnetic hybrid aerogels	104.6	74.6	Cr(VI) As(V)	833 426	[245]
Magnetic carbon aerogel	145.7	15.9	Cd(II)	143.88	[255]
Amphiprotic cellulose-mediated graphene oxide magnetic aerogels	-	8.61	Cu(II) Pb(II) Cd(II) Cr(III)	222.2 568.2 185.5 122.2	[249]

**Table 11:** Magnetic biochar-based adsorbents for various pollutants’ removal from contaminated water.

Adsorbent	Surface Area (m^2^ g^−1^)	Magnetic Saturation (emu g^−1^)	Pollutant	Adsorption Capacity (mg g^−1^)	References
Sludge-based magnetic biochar	20.19	25.60	Methylene blue	296.52	[263]
Magnetic wakame biochar nanocomposites	744.15	-	Methylene blue	450.92	[264]
Rice husk biochar-based magnetic nanocomposite	-	30.8	Crystal violet	185.6	[265]
Magnetic montmorillonite-biochar composite	67.77	35.10	Oxytetracycline	58.85	[266]
Magnetized biochar	-	44.1	Sulfadiazine Oxolinic acid	-	[267]
Magnetized biochar functionalized with TiO_2_	-	6.96	Sulfadiazine Oxolinic acid	-	[267]
Magnetized biochar functionalized with TiO_2_ and afterward magnetized by in situ	-	27.9	Sulfadiazine Oxolinic acid	-	[267]
Magnetized biochar functionalized with TiO_2_ and afterward magnetized by ex situ	-	33.5	Sulfadiazine Oxolinic acid	-	[267]
Magnetic Fe_3_O_4_ biochar	70.17	-	Tetracycline	29.4	[268]
Magnetic cobalt ferrite–biochar composite	83.23	39.11	Lomefloxacin hydrochloride	-	[269]
Magnetic CuZnFe_2_O_4_–biochar composite	61.5	37.6	Bisphenol A Sulfamethoxazole	-	[270]
Amino-modified rice bran biochar/MgFeAlO_4_ magnetic composites	34.13	19.78	Ni(II)	201.62	[271]
Amino hybrid biopolymer-decorated magnetic biochar composite—MTBC-2N	13.54	20.31	P	53.32	[272]
Amino hybrid biopolymer-decorated magnetic biochar composite—MTBC-4N	12.69	19.48	P	69.64	[272]
Halloysite and coconut shell–magnetic biochar composites	234–391	56.34–59.15	Pb(II)	415–680	[273]
Magnetic Zn/iron-based sludge/biochar composite	145.13	32.57	Cr(VI)	36.27	[274]
Magnetic greigite/biochar composites	10.2–17.6	-	Cr(VI)	-	[275]
Magnetic biochar composites	109.65	~9.45	U(VI)	52.63	[276]
Magnetic biogas residue-based biochar	79.64	39.96	Cu(II) Pb(II)	75.76 181.82	[277]
Biochar loaded with chitosan-stabilized ferrous sulfide nanoparticles	3.63–4.49	-	Cr(VI)	49.17–49.21	[278]

**Table 12:** Magnetic clay-based composite adsorbents for various pollutants’ removal from contaminated water.

Adsorbent	Surface Area (m^2^ g^−1^)	Magnetic Saturation (emu g^−1^)	Pollutant	Adsorption Capacity (mg g^−1^)	References
Fe_3_O_4_/kaolin magnetic nanocomposites	31.56	12.32	Direct red 23	22.88	[282]
Banded iron formation @bentonite	21.04	-	Crystal violet Acid red	117 91	[283]
Bacterial cellulose/attapulgite magnetic composites	197	16	Congo red	230	[284]
Magneto-carbon black-clay composite	-	-	Methylene blue	9.72	[285]
Clay/starch/MnFe_2_O_4_ magnetic nanocomposite	66.95	10.33	Sunset yellow Nile blue	79.81 86.78	[288]
Magnetic Fe_3_O_4_/zeolite NaA nanocomposite	~117	-	Methylene blue	40.36	[286]
Clay–magnetite nanocomposite	37.458	24.910	Naphthol blue-black	-	[289]
Graphene/magnetite/montmorillonite nanocomposite	97.916	49.95	Methylene blue	225.0	[287]
Magnetic montmorillonite composite	64.78	27.57	Enrofloxacin	-	[290]
Sodium dodecyl sulfate-modified BiOBr/magnetic bentonite	26.34	3.6	Tetracycline Ciprofloxacin	-	[291]
Iron oxide/hydrotalcite intercalated with dodecylsulfate/β-cyclodextrin magnetic organocomposite	-	-	Phenol P-nitrophenol P-cresol	216.08 255.63 272.48	[292]
Modified sepiolite clay loaded with Fe_3_O_4_	81.01	-	Atrazine	-	[293]
Magnetic sepiolite composite	81	26.22	Bisphenol A	-	[294]
Bacterial cellulose/attapulgite magnetic composites	197	16	Cr(VI) Cu(II) Pb(II)	91 70.5 67.8	[284]
Magneto-carbon black-clay composite	-	-	Cd(II)	8.83	[285]
EDTA-modified magnetic attapulgite chitosan gel beads	51.81	0.9	Pb(II) Cu(II) Ni(II)	368.32 267.94 220.31	[295]
Kaolin–bentonite–Fe_3_O_4_ composite	10	0.045	F^-^	-	[297]
Magnetic hydroxyapatite coated with manganese dioxide	131.826	11.713	Sr(II)	32.37	[298]
Magnetic bentonite/carboxymethyl chitosan/sodium alginate hydrogel beads	-	7.05	Cu(II)	56.79	[296]

**Table 13:** Comparative overview of surface areas and magnetic saturations for the described magnetic adsorbent categories.

Magnetic Composite Category	Surface Area Range (m^2^ g^−1^)	Magnetic Saturation Range (emu g^−1^)
Carbon nanotube-based	60–811	0.4–56
Graphene-based	32–213	7–62
Polymer-based	2–640	2.5–45
Hydrogel-based	0.3–26	0.18–32
MOF-based	2.6–1722	3–61
COF-based	56–2245	0.75–48
Silica-based	58–703	1.3–59
Aerogel-based	94–788	7.7–85
Biochar-based	3.6–744	7–59
Clay-based	10–197	0.05–50

## Data Availability

Not applicable.

## References

[1] Ingrao C., Strippoli R., Lagioia G., Huisingh D. (2023). Water scarcity in agriculture: An overview of causes, impacts and approaches for reducing the risks. Heliyon.

[2] Gleick P.H., Cooley H. (2021). Freshwater Scarcity. Annu. Rev. Environ. Resour..

[3] Alotaibi B.A., Baig M.B., Najim M.M.M., Shah A.A., Alamri Y.A. (2023). Water Scarcity Management to Ensure Food Scarcity through Sustainable Water Resources Management in Saudi Arabia. Sustainability.

[4] Ghernaout D. (2020). Water treatment challenges towards viruses removal. Open Access Libr. J..

[5] Topare N.S., Attar S.J., Manfe M.M. (2011). Sewage/wastewater treatment technologies: A review. Sci. Revs. Chem. Commun..

[6] Biswas P., Bose P., Tare V. (2007). Optimal choice of wastewater treatment train by multi-objective optimization. Eng. Optim..

[7] Boer J., Blaga P. (2016). Optimizing Production Costs by Redesigning the Treatment Process of the Industrial Waste Water. Procedia Technol..

[8] Dhangar K., Kumar M. (2020). Tricks and tracks in removal of emerging contaminants from the wastewater through hybrid treatment systems: A review. Sci. Total Environ..

[9] Bi J., Tao Q., Huang X., Wang J., Wang T., Hao H. (2021). Simultaneous decontamination of multi-pollutants: A promising approach for water remediation. Chemosphere.

[10] Rajasulochana P., Preethy V. (2016). Comparison on efficiency of various techniques in treatment of waste and sewage water—A comprehensive review. Resour. Effic. Technol..

[11] Ma Q., Li Y., Tan Y., Xu B., Cai J., Zhang Y., Wang Q., Wu Q., Yang B., Huang J. (2023). Recent Advances in Metal-Organic Framework (MOF)-Based Photocatalysts: Design Strategies and Applications in Heavy Metal Control. Molecules.

[12] Predescu A.M., Matei E., Berbecaru A.C., Râpă M., Sohaciu M.G., Predescu C., Vidu R. (2021). An Innovative Method of Converting Ferrous Mill Scale Wastes into Superparamagnetic Nanoadsorbents for Water Decontamination. Materials.

[13] Arshad A., Iqbal J., Mansoor Q. (2019). Graphene/Fe_3_O_4_ nanocomposite: Solar light driven Fenton like reaction for decontamination of water and inhibition of bacterial growth. Appl. Surf. Sci..

[14] Rossi L.M., Costa N.J.S., Silva F.P., Gonçalves R.V. (2013). Magnetic nanocatalysts: Supported metal nanoparticles for catalytic applications. Nanotechnol. Rev..

[15] Peralta M.E., Ocampo S., Funes I.G., Onaga Medina F., Parolo M.E., Carlos L. (2020). Nanomaterials with Tailored Magnetic Properties as Adsorbents of Organic Pollutants from Wastewaters. Inorganics.

[16] Mehta D., Mazumdar S., Singh S.K. (2015). Magnetic adsorbents for the treatment of water/wastewater—A review. J. Water Process Eng..

[17] Tatarchuk T., Soltys L., Macyk W. (2023). Magnetic adsorbents for removal of pharmaceuticals: A review of adsorption properties. J. Mol. Liq..

[18] Zeng X., Zhang G., Zhu J., Wu Z. (2022). Adsorption of heavy metal ions in water by surface functionalized magnetic composites: A review. Environ. Sci. Water Res. Technol..

[19] Fang K., Deng L., Yin J., Yang T., Li J., He W. (2022). Recent advances in starch-based magnetic adsorbents for the removal of contaminants from wastewater: A review. Int. J. Biol. Macromol..

[20] Sharma V.K., McDonald T.J., Kim H., Garg V.K. (2015). Magnetic graphene–carbon nanotube iron nanocomposites as adsorbents and antibacterial agents for water purification. Adv. Colloid Interface Sci..

[21] Agasti N., Gautam V., Priyanka, Manju, Pandey N., Genwa M., Meena P.L., Tandon S., Samantaray R. (2022). Carbon nanotube based magnetic composites for decontamination of organic chemical pollutants in water: A review. Appl. Surf. Sci. Adv..

[22] Mehmood A., Khan F.S.A., Mubarak N.M., Tan Y.H., Karri R.R., Khalid M., Walvekar R., Abdullah E.C., Nizamuddin S., Mazari S.A. (2021). Magnetic nanocomposites for sustainable water purification—A comprehensive review. Environ. Sci. Pollut. Res..

[23] Nisticò R. (2017). Magnetic materials and water treatments for a sustainable future. Res. Chem. Intermed..

[24] Bruckmann F.S., Schnorr C., Oviedo L.R., Knani S., Silva L.F.O., Silva W.L., Dotto G.L., Bohn Rhoden C.R. (2022). Adsorption and Photocatalytic Degradation of Pesticides into Nanocomposites: A Review. Molecules.

[25] Yu M., Wang L., Hu L., Li Y., Luo D., Mei S. (2019). Recent applications of magnetic composites as extraction adsorbents for determination of environmental pollutants. TrAC Trends Anal. Chem..

[26] Abdullah N.H., Shameli K., Abdullah E.C., Abdullah L.C. (2019). Solid matrices for fabrication of magnetic iron oxide nanocomposites: Synthesis, properties, and application for the adsorption of heavy metal ions and dyes. Compos. Part B Eng..

[27] Sharma A., Mangla D., Shehnaz, Chaudhry S.A. (2022). Recent advances in magnetic composites as adsorbents for wastewater remediation. J. Environ. Manag..

[28] Xu W., Yang T., Liu S., Du L., Chen Q., Li X., Dong J., Zhang Z., Lu S., Gong Y. (2022). Insights into the Synthesis, types and application of iron Nanoparticles: The overlooked significance of environmental effects. Environ. Int..

[29] Iqbal A., Jalees M.I., Farooq M.U., Cevik E., Bozkurt A. (2022). Superfast adsorption and high-performance tailored membrane filtration by engineered Fe-Ni-Co nanocomposite for simultaneous removal of surface water pollutants. Colloids Surf. A Physicochem. Eng. Asp..

[30] Dong H., Jiang Z., Deng J., Zhang C., Cheng Y., Hou K., Zhang L., Tang L., Zeng G. (2018). Physicochemical transformation of Fe/Ni bimetallic nanoparticles during aging in simulated groundwater and the consequent effect on contaminant removal. Water Res..

[31] Sharma R.K., Arora B., Sharma S., Dutta S., Sharma A., Yadav S., Solanki K. (2020). In Situ hydroxyl radical generation using the synergism of the Co–Ni bimetallic centres of a developed nanocatalyst with potent efficiency for degrading toxic water pollutants. Mater. Chem. Front..

[32] Deshpande N.G., Ahn C.H., Kim D.S., Jung S.H., Kim Y.B., Cho H.K. (2020). Bifunctional reusable Co_0_._5_Ni_0_._5_Fe_2_O_4_ nanoparticle-grafted carbon nanotubes for aqueous dye removal from contaminated water. Catal. Sci. Technol..

[33] Zhang X., Cui C., Wang Y., Chang J., Ma D., Wang J. (2020). An efficient method for removal of pentachlorophenol using adsorption and microwave regeneration with different magnetic carbon nanotubes. Water Sci. Technol..

[34] Xiao R., Abdu H.I., Wei L., Wang T., Huo S., Chen J., Lu X. (2020). Fabrication of magnetic trimetallic metal–organic frameworks for the rapid removal of tetracycline from water. Analyst.

[35] Kotal M., Sharma A., Jakhar S., Mishra V., Roy S., Sahoo S.C., Sharma H.K., Mehta S.K. (2020). Graphene-Templated Cobalt Nanoparticle Embedded Nitrogen-Doped Carbon Nanotubes for Efficient Visible-Light Photocatalysis. Cryst. Growth Des..

[36] Jin L., Zhao X., Qian X., Dong M. (2018). Nickel nanoparticles encapsulated in porous carbon and carbon nanotube hybrids from bimetallic metal-organic-frameworks for highly efficient adsorption of dyes. J. Colloid Interface Sci..

[37] Ma J., Ma Y., Yu F. (2018). A Novel One-Pot Route for Large-Scale Synthesis of Novel Magnetic CNTs/Fe@C Hybrids and Their Applications for Binary Dye Removal. ACS Sustain. Chem. Eng..

[38] Abou Hammad A.B., El Nahwary A.M., Hemdan B.A., Abia A.L.K. (2020). Nanoceramics and novel functionalized silicate-based magnetic nanocomposites as substitutional disinfectants for water and wastewater purification. Environ. Sci. Pollut. Res..

[39] Mostafapour F.K., Miri A., Khatibi A., Balarak D., Kyzas G.Z. (2023). Survey of Fe_3_O_4_ Magnetic Nanoparticles Modified with Sodium Dodecyl Sulfate for Removal P-Cresol and Pyrocatechol from Aqueous Solutions. Biointerface Res. Appl. Chem..

[40] Mohamed G., Ashraf A., Mohamed A. (2022). Low-Temperature Adsorption Study of Carbon Dioxide on Porous Magnetite Nanospheres Iron Oxide. Biointerface Res. Appl. Chem..

[41] Shokrollahi H. (2017). A review of the magnetic properties, synthesis methods and applications of maghemite. J. Magn. Magn. Mater..

[42] Niculescu A.-G., Chircov C., Grumezescu A.M. (2022). Magnetite nanoparticles: Synthesis methods—A comparative review. Methods.

[43] Fatima G., Bibi I., Majid F., Kamal S., Nouren S., Ghafoor A., Raza Q., Al-Mijalli S.H., Alnafisi N.M., Iqbal M. (2023). Mn-doped BaFe_12_O_19_ nanoparticles synthesis via micro-emulsion route: Solar light-driven photo-catalytic degradation of CV, MG and RhB dyes and antibacterial activity. Mater. Res. Bull..

[44] Misbah, Bibi I., Majid F., Kamal S., Jilani K., Taj B., Nazeer Z., Iqbal M. (2022). Enhanced visible light-driven photocatalytic degradation of crystal violet dye using Cr doped BaFe_12_O_19_ prepared via facile micro-emulsion route. J. Saudi Chem. Soc..

[45] Zhou P., Wang Y., Yan X., Gan Y., Xia C., Xu Y., Xie M. (2024). Nitrogen-defect-modified g-C_3_N_4_/BaFe_12_O_19_ S-scheme heterojunction photocatalyst with enhanced advanced oxidation technology synergistic photothermal degradation ability of antibiotic: Insights into performance, electron transfer pathways and toxicity. Appl. Catal. B Environ..

[46] Wang H., Xu L., Liu C., Jiang Z., Feng Q., Wu T., Wang R. (2020). A novel magnetic photocatalyst Bi_3_O_4_Cl/SrFe_12_O_19_: Fabrication, characterization and its photocatalytic activity. Ceram. Int..

[47] Lahijani B., Hedayati K., Goodarzi M. (2018). Magnetic PbFe_12_O_19_-TiO_2_ nanocomposites and their photocatalytic performance in the removal of toxic pollutants. Main Group Met. Chem..

[48] Chen X., Wang S., Gao H., Yang H., Fang L., Chen X., Tang S., Yu C., Li D. (2022). A novel lead hexagonal ferrite (PbFe_12_O_19_) magnetic separation catalyst with excellent ultrasonic catalytic activity. J. Sol-Gel Sci. Technol..

[49] Zhao Y., Liu Y., Xu H., Fan Q., Zhu C., Liu J., Zhu M., Wang X., Niu A. (2023). Preparation and Application of Magnetic Composites Using Controllable Assembly for Use in Water Treatment: A Review. Molecules.

[50] Jangra A., Singh J., Kumar J., Rani K., Kumar P., Kumar S., Singh D., Kumar R. (2023). Dye Elimination by Surface-Functionalized Magnetite Nanoparticles: Kinetic and Isotherm Studies. Biointerface Res. Appl. Chem..

[51] Jain N., Jee Kanu N. (2021). The potential application of carbon nanotubes in water Treatment: A state-of-the-art-review. Mater. Today Proc..

[52] Kumar R., Rauwel P., Rauwel E. (2021). Nanoadsorbants for the Removal of Heavy Metals from Contaminated Water: Current Scenario and Future Directions. Processes.

[53] Arora B., Attri P. (2020). Carbon Nanotubes (CNTs): A Potential Nanomaterial for Water Purification. J. Compos. Sci..

[54] Rao G.P., Lu C., Su F. (2007). Sorption of divalent metal ions from aqueous solution by carbon nanotubes: A review. Sep. Purif. Technol..

[55] Mubarak N.M., Sahu J.N., Abdullah E.C., Jayakumar N.S. (2014). Removal of heavy metals from wastewater using carbon nanotubes. Sep. Purif. Rev..

[56] Li Y.-H., Ding J., Luan Z., Di Z., Zhu Y., Xu C., Wu D., Wei B. (2003). Competitive adsorption of Pb^2+^, Cu^2+^ and Cd^2+^ ions from aqueous solutions by multiwalled carbon nanotubes. Carbon.

[57] Islam M.R., Ferdous M., Sujan M.I., Mao X., Zeng H., Azam M.S. (2020). Recyclable Ag-decorated highly carbonaceous magnetic nanocomposites for the removal of organic pollutants. J. Colloid Interface Sci..

[58] Çalımlı M.H. (2021). Magnetic nanocomposite cobalt-multiwalled carbon nanotube and adsorption kinetics of methylene blue using an ultrasonic batch. Int. J. Environ. Sci. Technol..

[59] Ye J., Li C., Yan Y. (2020). Core-shell ZIF-67/ZIF-8-derived sea urchin-like cobalt/nitrogen Co-doped carbon nanotube hollow frameworks for ultrahigh adsorption and catalytic activities. J. Taiwan Inst. Chem. Eng..

[60] Ahamad T., Naushad M., Eldesoky G.E., Al-Saeedi S.I., Nafady A., Al-Kadhi N.S., Al-Muhtaseb A.a.H., Khan A.A., Khan A. (2019). Effective and fast adsorptive removal of toxic cationic dye (MB) from aqueous medium using amino-functionalized magnetic multiwall carbon nanotubes. J. Mol. Liq..

[61] Liu Y., Guo L., Huang H., Dou J., Huang Q., Gan D., Chen J., Li Y., Zhang X., Wei Y. (2019). Facile preparation of magnetic composites based on carbon nanotubes: Utilization for removal of environmental pollutants. J. Colloid Interface Sci..

[62] Bhakta A.K., Kumari S., Hussain S., Martis P., Mascarenhas R.J., Delhalle J., Mekhalif Z. (2019). Synthesis and characterization of maghemite nanocrystals decorated multi-wall carbon nanotubes for methylene blue dye removal. J. Mater. Sci..

[63] Hosseinzadeh S., Hosseinzadeh H., Pashaei S., Khodaparast Z. (2018). Synthesis of magnetic functionalized MWCNT nanocomposite through surface RAFT co-polymerization of acrylic acid and N-isopropyl acrylamide for removal of cationic dyes from aqueous solutions. Ecotoxicol. Environ. Saf..

[64] Feng K., Song B., Li X., Liao F., Gong J. (2019). Enhanced photocatalytic performance of magnetic multi-walled carbon nanotubes/cerium dioxide nanocomposite. Ecotoxicol. Environ. Saf..

[65] Jelokhani F., Sheibani S., Ataie A. (2020). Adsorption and photocatalytic characteristics of cobalt ferrite-reduced graphene oxide and cobalt ferrite-carbon nanotube nanocomposites. J. Photochem. Photobiol. A Chem..

[66] Hezam F.A., Nur O., Mustafa M.A. (2020). Synthesis, structural, optical and magnetic properties of NiFe_2_O_4_/MWCNTs/ZnO hybrid nanocomposite for solar radiation driven photocatalytic degradation and magnetic separation. Colloids Surf. A Physicochem. Eng. Asp..

[67] Kang J., Zhang H., Duan X., Sun H., Tan X., Liu S., Wang S. (2019). Magnetic Ni-Co alloy encapsulated N-doped carbon nanotubes for catalytic membrane degradation of emerging contaminants. Chem. Eng. J..

[68] Hu C., Le A.T., Pung S.Y., Stevens L., Neate N., Hou X., Grant D., Xu F. (2021). Efficient dye-removal via Ni-decorated graphene oxide-carbon nanotube nanocomposites. Mater. Chem. Phys..

[69] Zhu K., Bin Q., Shen Y., Huang J., He D., Chen W. (2020). In-situ formed N-doped bamboo-like carbon nanotubes encapsulated with Fe nanoparticles supported by biochar as highly efficient catalyst for activation of persulfate (PS) toward degradation of organic pollutants. Chem. Eng. J..

[70] Yang G., Li Y., Yang S., Liao J., Cai X., Gao Q., Fang Y., Peng F., Zhang S. (2021). Surface oxidized nano-cobalt wrapped by nitrogen-doped carbon nanotubes for efficient purification of organic wastewater. Sep. Purif. Technol..

[71] Tian X., Xiao L. (2020). FeOx/MnOy modified oxidized carbon nanotubes as peroxymonosulfate activator for organic pollutants degradation. J. Colloid Interface Sci..

[72] Ma T., Wu Y., Liu N., Wu Y. (2020). Iron manganese Oxide Modified Multi-walled Carbon Nanotube as Efficient Adsorbent for Removal of Organic Dyes: Performance, Kinetics and Mechanism Studies. J. Inorg. Organomet. Polym. Mater..

[73] Dastkhoon M., Ghaedi M., Asfaram A., Jannesar R., Sadeghfar F. (2018). Magnetic based nanocomposite sorbent combination with ultrasound assisted for solid-phase microextraction of Azure II in water samples prior to its determination spectrophotometric. J. Colloid Interface Sci..

[74] Foroutan R., Peighambardoust S.J., Esvandi Z., Khatooni H., Ramavandi B. (2021). Evaluation of two cationic dyes removal from aqueous environments using CNT/MgO/CuFe_2_O_4_ magnetic composite powder: A comparative study. J. Environ. Chem. Eng..

[75] Duman S., Erbas Z., Soylak M. (2020). Ultrasound-assisted magnetic solid phase microextraction of patent blue V on magnetic multiwalled carbon nanotubes prior to its spectrophotometric determination. Microchem. J..

[76] Tavakoli M., Safa F., Abedinzadeh N. (2019). Binary nanocomposite of Fe3O4/MWCNTs for adsorption of Reactive Violet 2: Taguchi design, kinetics and equilibrium isotherms. Fuller. Nanotub. Carbon Nanostruct..

[77] Zhang Z., Chen H., Wu W., Pang W., Yan G. (2019). Efficient removal of Alizarin Red S from aqueous solution by polyethyleneimine functionalized magnetic carbon nanotubes. Bioresour. Technol..

[78] Nas M.S., Kuyuldar E., Demirkan B., Calimli M.H., Demirbaş O., Sen F. (2019). Magnetic nanocomposites decorated on multiwalled carbon nanotubefor removal of Maxilon Blue 5G using the sono-Fenton method. Sci. Rep..

[79] Deng Y., Ok Y.S., Mohan D., Pittman C.U., Dou X. (2019). Carbamazepine removal from water by carbon dot-modified magnetic carbon nanotubes. Environ. Res..

[80] Awfa D., Ateia M., Fujii M., Yoshimura C. (2019). Novel Magnetic Carbon Nanotube-TiO_2_ Composites for Solar Light Photocatalytic Degradation of Pharmaceuticals in the Presence of Natural Organic Matter. J. Water Process Eng..

[81] Zhang R., Wang S., Yang Y., Deng Y., Li D., Su P., Yang Y. (2018). Modification of polydopamine-coated Fe_3_O_4_ nanoparticles with multi-walled carbon nanotubes for magnetic-μ-dispersive solid-phase extraction of antiepileptic drugs in biological matrices. Anal. Bioanal. Chem..

[82] Alizadeh Fard M., Barkdoll B. (2018). Using recyclable magnetic carbon nanotube to remove micropollutants from aqueous solutions. J. Mol. Liq..

[83] Xiong W., Zeng G., Yang Z., Zhou Y., Zhang C., Cheng M., Liu Y., Hu L., Wan J., Zhou C. (2018). Adsorption of tetracycline antibiotics from aqueous solutions on nanocomposite multi-walled carbon nanotube functionalized MIL-53(Fe) as new adsorbent. Sci. Total Environ..

[84] Xiong W., Zeng Z., Li X., Zeng G., Xiao R., Yang Z., Zhou Y., Zhang C., Cheng M., Hu L. (2018). Multi-walled carbon nanotube/amino-functionalized MIL-53(Fe) composites: Remarkable adsorptive removal of antibiotics from aqueous solutions. Chemosphere.

[85] Chen S., Zang Z., Zhang S., Ouyang G., Han R. (2021). Preparation of MIL-100(Fe) and multi-walled carbon nanotubes nanocomposite with high adsorption capacity towards Oxytetracycline from solution. J. Environ. Chem. Eng..

[86] Zhao Y., Tang J.J., Motavalizadehkakhky A., Kakooei S., Sadeghzadeh S.M. (2019). Synthesis and characterization of a novel CNT-FeNi_3_/DFNS/Cu(ii) magnetic nanocomposite for the photocatalytic degradation of tetracycline in wastewater. RSC Adv..

[87] Foroutan R., Peighambardoust S.J., Latifi P., Ahmadi A., Alizadeh M., Ramavandi B. (2021). Carbon nanotubes/β-cyclodextrin/MnFe_2_O_4_ as a magnetic nanocomposite powder for tetracycline antibiotic decontamination from different aqueous environments. J. Environ. Chem. Eng..

[88] Shang Y., Chen C., Zhang P., Yue Q., Li Y., Gao B., Xu X. (2019). Removal of sulfamethoxazole from water via activation of persulfate by Fe_3_C@NCNTs including mechanism of radical and nonradical process. Chem. Eng. J..

[89] Nawaz M., Shahzad A., Tahir K., Kim J., Moztahida M., Jang J., Alam M.B., Lee S.-H., Jung H.-Y., Lee D.S. (2020). Photo-Fenton reaction for the degradation of sulfamethoxazole using a multi-walled carbon nanotube-NiFe_2_O_4_ composite. Chem. Eng. J..

[90] Yanan S., Xing X., Yue Q., Gao B., Li Y. (2020). Nitrogen-doped carbon nanotubes encapsulating Fe/Zn nanoparticles as a persulfate activator for sulfamethoxazole degradation: Role of encapsulated bimetallic nanoparticles and nonradical reaction. Environ. Sci. Nano.

[91] Sadeghi M., Mehdinejad M.H., Mengelizadeh N., Mahdavi Y., Pourzamani H., Hajizadeh Y., Zare M.R. (2019). Degradation of diclofenac by heterogeneous electro-Fenton process using magnetic single-walled carbon nanotubes as a catalyst. J. Water Process Eng..

[92] Ulusoy H.İ., Yılmaz E., Soylak M. (2019). Magnetic solid phase extraction of trace paracetamol and caffeine in synthetic urine and wastewater samples by a using core shell hybrid material consisting of graphene oxide/multiwalled carbon nanotube/Fe_3_O_4_/SiO_2_. Microchem. J..

[93] Barrios-Bermúdez N., González-Avendaño M., Lado-Touriño I., Cerpa-Naranjo A., Rojas-Cervantes M.L. (2020). Fe-Cu Doped Multiwalled Carbon Nanotubes for Fenton-like Degradation of Paracetamol Under Mild Conditions. Nanomaterials.

[94] Lung I., Soran M.-L., Stegarescu A., Opris O., Gutoiu S., Leostean C., Lazar M.D., Kacso I., Silipas T.-D., Porav A.S. (2021). Evaluation of CNT-COOH/MnO_2_/Fe_3_O_4_ nanocomposite for ibuprofen and paracetamol removal from aqueous solutions. J. Hazard. Mater..

[95] Quan X., Sun Z., Xu J., Liu S., Han Y., Xu Y., Meng H., Wu J., Zhang X. (2020). Construction of an Aminated MIL-53(Al)-Functionalized Carbon Nanotube for the Efficient Removal of Bisphenol AF and Metribuzin. Inorg. Chem..

[96] Duman O., Özcan C., Gürkan Polat T., Tunç S. (2019). Carbon nanotube-based magnetic and non-magnetic adsorbents for the high-efficiency removal of diquat dibromide herbicide from water: OMWCNT, OMWCNT-Fe_3_O_4_ and OMWCNT-κ-carrageenan-Fe_3_O_4_ nanocomposites. Environ. Pollut..

[97] Liu G., Li L., Huang X., Zheng S., Xu X., Liu Z., Zhang Y., Wang J., Lin H., Xu D. (2018). Adsorption and removal of organophosphorus pesticides from environmental water and soil samples by using magnetic multi-walled carbon nanotubes @ organic framework ZIF-8. J. Mater. Sci..

[98] Li S., Li Z., Ke B., He Z., Cui Y., Pan Z., Li D., Huang S., Lai C., Su J. (2019). Magnetic multi-walled carbon nanotubes modified with polyaluminium chloride for removal of humic acid from aqueous solution. J. Mol. Liq..

[99] Mirzaee S.A., Jaafarzadeh N., Gomes H.T., Jorfi S., Ahmadi M. (2019). Magnetic titanium/carbon nanotube nanocomposite catalyst for oxidative degradation of Bisphenol A from high saline polycarbonate plant effluent using catalytic wet peroxide oxidation. Chem. Eng. J..

[100] Mohammadi A.A., Dehghani M.H., Mesdaghinia A., Yaghmaian K., Es’haghi Z. (2020). Adsorptive removal of endocrine disrupting compounds from aqueous solutions using magnetic multi-wall carbon nanotubes modified with chitosan biopolymer based on response surface methodology: Functionalization, kinetics, and isotherms studies. Int. J. Biol. Macromol..

[101] Huang Y., Zhang W., Bai M., Huang X. (2020). One-pot fabrication of magnetic fluorinated carbon nanotubes adsorbent for efficient extraction of perfluoroalkyl carboxylic acids and perfluoroalkyl sulfonic acids in environmental water samples. Chem. Eng. J..

[102] Pourzamani H., Hashemi M., Bina B., Rashidi A., Amin Mohammad M., Parastar S. (2018). Toluene Removal from Aqueous Solutions Using Single-Wall Carbon Nanotube and Magnetic Nanoparticle–Hybrid Adsorbent. J. Environ. Eng..

[103] Yang Y., Zhang H., Huang H., Yan Y., Zhang X. (2021). Degradation of m-cresol over iron loaded carbon nanotube microfibrous composite: Kinetic optimization and deactivation study. Sep. Purif. Technol..

[104] Jalilian N., Ebrahimzadeh H., Asgharinezhad A.A. (2019). Preparation of magnetite/multiwalled carbon nanotubes/metal-organic framework composite for dispersive magnetic micro solid phase extraction of parabens and phthalate esters from water samples and various types of cream for their determination with liquid chromatography. J. Chromatogr. A.

[105] Zhang H., Chen S., Zhang H., Fan X., Gao C., Yu H., Quan X. (2019). Carbon nanotubes-incorporated MIL-88B-Fe as highly efficient Fenton-like catalyst for degradation of organic pollutants. Front. Environ. Sci. Eng..

[106] Ashtiani S., Khoshnamvand M., Shaliutina-Kolešová A., Bouša D., Sofer Z., Friess K. (2020). Co_0_._5_Ni_0_._5_FeCrO_4_ spinel nanoparticles decorated with UiO-66-based metal-organic frameworks grafted onto GO and O-SWCNT for gas adsorption and water purification. Chemosphere.

[107] Bhaduri B., Engel M., Polubesova T., Wu W., Xing B., Chefetz B. (2018). Dual functionality of an Ag-Fe_3_O_4_-carbon nanotube composite material: Catalytic reduction and antibacterial activity. J. Environ. Chem. Eng..

[108] Wang D., Liu J., Xi J., Jiang J., Bai Z. (2019). Pd-Fe dual-metal nanoparticles confined in the interface of carbon nanotubes/N-doped carbon for excellent catalytic performance. Appl. Surf. Sci..

[109] Zhang X., Cui C., Zheng Q., Wang Y., Chang J., Wang S. (2021). Development of highly efficient and reusable magnetic nitrogen-doped carbon nanotubes for chlorophenol removal. Environ. Sci. Pollut. Res..

[110] Khan A.A.P., Singh P., Raizada P., Asiri A.M. (2020). Synthesis of magnetically separable Bi_2_O_2_CO_3_/carbon nanotube/ZnFe_2_O_4_ as Z-scheme heterojunction with enhanced photocatalytic activity for water purification. J. Sol-Gel Sci. Technol..

[111] Raizada P., Aslam Parwaz Khan A., Singh P. (2020). Construction of carbon nanotube mediated Fe doped graphitic carbon nitride and Ag_3_VO_4_ based Z-scheme heterojunction for H_2_O_2_ assisted 2,4 dimethyl phenol photodegradation. Sep. Purif. Technol..

[112] Deng D., He Y., Li M., Huang L., Zhang J. (2021). Preparation of multi-walled carbon nanotubes based magnetic multi-template molecularly imprinted polymer for the adsorption of phthalate esters in water samples. Environ. Sci. Pollut. Res..

[113] Han Z., Huang L., Qu H., Wang Y., Zhang Z., Rong Q., Sang Z., Wang Y., Kipper M.J., Tang J. (2021). A review of performance improvement strategies for graphene oxide-based and graphene-based membranes in water treatment. J. Mater. Sci..

[114] Lingamdinne L.P., Koduru J.R., Karri R.R. (2019). A comprehensive review of applications of magnetic graphene oxide based nanocomposites for sustainable water purification. J. Environ. Manag..

[115] Xie A., Cui J., Yang J., Chen Y., Lang J., Li C., Yan Y., Dai J. (2020). Graphene oxide/Fe(III)-based metal-organic framework membrane for enhanced water purification based on synergistic separation and photo-Fenton processes. Appl. Catal. B Environ..

[116] Abbasi S., Ahmadpoor F., Imani M., Ekrami-Kakhki M.-S. (2020). Synthesis of magnetic Fe_3_O_4_@ZnO@graphene oxide nanocomposite for photodegradation of organic dye pollutant. Int. J. Environ. Anal. Chem..

[117] Foroutan R., Mohammadi R., MousaKhanloo F., Sahebi S., Ramavandi B., Kumar P.S., Vardhan K.H. (2020). Performance of montmorillonite/graphene oxide/CoFe2O4 as a magnetic and recyclable nanocomposite for cleaning methyl violet dye-laden wastewater. Adv. Powder Technol..

[118] Bayantong A.R.B., Shih Y.-J., Ong D.C., Abarca R.R.M., Dong C.-D., de Luna M.D.G. (2021). Adsorptive removal of dye in wastewater by metal ferrite-enabled graphene oxide nanocomposites. Chemosphere.

[119] Li Y., Dong X., Zhao L. (2021). Application of magnetic chitosan nanocomposites modified by graphene oxide and polyethyleneimine for removal of toxic heavy metals and dyes from water. Int. J. Biol. Macromol..

[120] Qiao D., Li Z., Duan J., He X. (2020). Adsorption and photocatalytic degradation mechanism of magnetic graphene oxide/ZnO nanocomposites for tetracycline contaminants. Chem. Eng. J..

[121] Karimi-Maleh H., Shafieizadeh M., Taher M.A., Opoku F., Kiarii E.M., Govender P.P., Ranjbari S., Rezapour M., Orooji Y. (2020). The role of magnetite/graphene oxide nano-composite as a high-efficiency adsorbent for removal of phenazopyridine residues from water samples, an experimental/theoretical investigation. J. Mol. Liq..

[122] Prarat P., Hongsawat P., Punyapalakul P. (2020). Amino-functionalized mesoporous silica-magnetic graphene oxide nanocomposites as water-dispersible adsorbents for the removal of the oxytetracycline antibiotic from aqueous solutions: Adsorption performance, effects of coexisting ions, and natural organic matter. Environ. Sci. Pollut. Res..

[123] Ninwiwek N., Hongsawat P., Punyapalakul P., Prarat P. (2019). Removal of the antibiotic sulfamethoxazole from environmental water by mesoporous silica-magnetic graphene oxide nanocomposite technology: Adsorption characteristics, coadsorption and uptake mechanism. Colloids Surf. A Physicochem. Eng. Asp..

[124] Fakhri H., Farzadkia M., Boukherroub R., Srivastava V., Sillanpää M. (2020). Design and preparation of core-shell structured magnetic graphene oxide@MIL-101(Fe): Photocatalysis under shell to remove diazinon and atrazine pesticides. Sol. Energy.

[125] Katubi K.M., Alsaiari N.S., Alzahrani F.M., Siddeeg S.M., Tahoon M.A. (2021). Synthesis of Manganese Ferrite/Graphene Oxide Magnetic Nanocomposite for Pollutants Removal from Water. Processes.

[126] Bi J., Huang X., Wang J., Wang T., Wu H., Yang J., Lu H., Hao H. (2019). Oil-phase cyclic magnetic adsorption to synthesize Fe_3_O_4_@C@TiO_2_-nanotube composites for simultaneous removal of Pb(II) and Rhodamine B. Chem. Eng. J..

[127] Ain Q.-U., Farooq M.U., Jalees M.I. (2020). Application of Magnetic Graphene Oxide for Water Purification: Heavy Metals Removal and Disinfection. J. Water Process Eng..

[128] Bao S., Yang W., Wang Y., Yu Y., Sun Y. (2020). One-pot synthesis of magnetic graphene oxide composites as an efficient and recoverable adsorbent for Cd(II) and Pb(II) removal from aqueous solution. J. Hazard. Mater..

[129] Suo L., Dong X., Gao X., Xu J., Huang Z., Ye J., Lu X., Zhao L. (2019). Silica-coated magnetic graphene oxide nanocomposite based magnetic solid phase extraction of trace amounts of heavy metals in water samples prior to determination by inductively coupled plasma mass spectrometry. Microchem. J..

[130] Hassan A.M., Wan Ibrahim W.A., Bakar M.B., Sanagi M.M., Sutirman Z.A., Nodeh H.R., Mokhter M.A. (2020). New effective 3-aminopropyltrimethoxysilane functionalized magnetic sporopollenin-based silica coated graphene oxide adsorbent for removal of Pb(II) from aqueous environment. J. Environ. Manag..

[131] Marques Neto J.d.O., Bellato C.R., Silva D.d.C. (2019). Iron oxide/carbon nanotubes/chitosan magnetic composite film for chromium species removal. Chemosphere.

[132] Naicker C., Nombona N., Van Zyl W.E. (2020). Fabrication of novel magnetic chitosan/graphene-oxide/metal oxide nanocomposite beads for Cr(VI) adsorption. Chem. Pap..

[133] Neolaka Y.A.B., Lawa Y., Naat J.N., Riwu A.A.P., Iqbal M., Darmokoesoemo H., Kusuma H.S. (2020). The adsorption of Cr(VI) from water samples using graphene oxide-magnetic (GO-Fe_3_O_4_) synthesized from natural cellulose-based graphite (kusambi wood or Schleichera oleosa): Study of kinetics, isotherms and thermodynamics. J. Mater. Res. Technol..

[134] Anush S.M., Chandan H.R., Gayathri B.H., Asma, Manju N., Vishalakshi B., Kalluraya B. (2020). Graphene oxide functionalized chitosan-magnetite nanocomposite for removal of Cu(II) and Cr(VI) from waste water. Int. J. Biol. Macromol..

[135] Fayazi M. (2020). Removal of mercury(II) from wastewater using a new and effective composite: Sulfur-coated magnetic carbon nanotubes. Environ. Sci. Pollut. Res..

[136] Lingamdinne L.P., Lee S., Choi J.-S., Lebaka V.R., Durbaka V.R.P., Koduru J.R. (2021). Potential of the magnetic hollow sphere nanocomposite (graphene oxide-gadolinium oxide) for arsenic removal from real field water and antimicrobial applications. J. Hazard. Mater..

[137] Koduru J.R., Karri R.R., Mubarak N.M., Inamuddin T.S., Kumar M.R., Asiri A.M. (2019). Smart Materials, Magnetic Graphene Oxide-Based Nanocomposites for Sustainable Water Purification. Sustainable Polymer Composites and Nanocomposites.

[138] Wang Y., Ren N., Xi J., Liu Y., Kong T., Chen C., Xie Y., Duan X., Wang S. (2021). Mechanistic Investigations of the Pyridinic N–Co Structures in Co Embedded N-Doped Carbon Nanotubes for Catalytic Ozonation. ACS EST Eng..

[139] Chen H., Huang C., Zhang W., Ding Q., Gao J., Zhang L. (2019). Ultrastable nitrogen-doped carbon nanotube encapsulated cobalt nanoparticles for magnetic solid-phase extraction of okadaic acid from aquatic samples. J. Chromatogr. A.

[140] Zhou Q., Yuan Y., Sun Y., Sheng X., Tong Y. (2021). Magnetic solid phase extraction of heterocyclic aromatic hydrocarbons from environmental water samples with multiwalled carbon nanotube modified magnetic polyamido-amine dendrimers prior to gas chromatography-triple quadrupole mass spectrometer. J. Chromatogr. A.

[141] Alipoori S., Rouhi H., Linn E., Stumpfl H., Mokarizadeh H., Esfahani M.R., Koh A., Weinman S.T., Wujcik E.K. (2021). Polymer-Based Devices and Remediation Strategies for Emerging Contaminants in Water. ACS Appl. Polym. Mater..

[142] Kumar M., Dosanjh H.S., Singh J., Monir K., Singh H. (2020). Review on magnetic nanoferrites and their composites as alternatives in waste water treatment: Synthesis, modifications and applications. Environ. Sci. Water Res. Technol..

[143] Karthik V., Dhivya Dharshini G., Senthil Kumar P., Kiruthika S., Rangasamy G., Periyasamy S., Senthil Rathi B. (2023). Ferrite-Supported Nanocomposite Polymers for Emerging Organic and Inorganic Pollutants Removal from Wastewater: A Review. Ind. Eng. Chem. Res..

[144] dos Santos J.M.N., Pereira C.R., Foletto E.L., Dotto G.L. (2019). Alternative synthesis for ZnFe_2_O_4_/chitosan magnetic particles to remove diclofenac from water by adsorption. Int. J. Biol. Macromol..

[145] Kumar M., Dosanjh H.S., Singh H. (2018). Magnetic Zinc Ferrite–Chitosan Bio-Composite: Synthesis, Characterization and Adsorption Behavior Studies for Cationic Dyes in Single and Binary Systems. J. Inorg. Organomet. Polym. Mater..

[146] Simonescu C.M., Tătăruş A., Culiţă D.C., Stănică N., Ionescu I.A., Butoi B., Banici A.-M. (2021). Comparative Study of CoFe_2_O_4_ Nanoparticles and CoFe_2_O_4_-Chitosan Composite for Congo Red and Methyl Orange Removal by Adsorption. Nanomaterials.

[147] Liang X.X., Omer A.M., Hu Z., Wang Y., Yu D., Ouyang X. (2019). Efficient adsorption of diclofenac sodium from aqueous solutions using magnetic amine-functionalized chitosan. Chemosphere.

[148] Kumar M., Dosanjh H.S., Singh H. (2019). Biopolymer modified transition metal spinel ferrites for removal of fluoride ions from water. Environ. Nanotechnol. Monit. Manag..

[149] Alzahrani F.M., Alsaiari N.S., Katubi K.M., Amari A., Ben Rebah F., Tahoon M.A. (2021). Synthesis of Polymer-Based Magnetic Nanocomposite for Multi-Pollutants Removal from Water. Polymers.

[150] Zhou Q., Wang Y., Xiao J., Fan H., Chen C. (2019). Preparation and characterization of magnetic nanomaterial and its application for removal of polycyclic aromatic hydrocarbons. J. Hazard. Mater..

[151] Wu H., Zhang H., Zhang W., Yang X., Zhou H., Pan Z., Wang D. (2019). Preparation of magnetic polyimide@ Mg-Fe layered double hydroxides core-shell composite for effective removal of various organic contaminants from aqueous solution. Chemosphere.

[152] Ali I., Peng C., Naz I., Lin D., Saroj D.P., Ali M. (2019). Development and application of novel bio-magnetic membrane capsules for the removal of the cationic dye malachite green in wastewater treatment. RSC Adv..

[153] Rossatto D.L., Netto M.S., Jahn S.L., Mallmann E.S., Dotto G.L., Foletto E.L. (2020). Highly efficient adsorption performance of a novel magnetic geopolymer/Fe_3_O_4_ composite towards removal of aqueous acid green 16 dye. J. Environ. Chem. Eng..

[154] Jodeh S., Hamed O., Melhem A., Salghi R., Jodeh D., Azzaoui K., Benmassaoud Y., Murtada K. (2018). Magnetic nanocellulose from olive industry solid waste for the effective removal of methylene blue from wastewater. Environ. Sci. Pollut. Res..

[155] Liu D., Huang Z., Li M., Sun P., Yu T., Zhou L. (2019). Novel porous magnetic nanospheres functionalized by β-cyclodextrin polymer and its application in organic pollutants from aqueous solution. Environ. Pollut..

[156] Ahamad T., Naushad M., Ruksana, Alshehri S.M. (2019). Ultra-fast spill oil recovery using a mesoporous lignin based nanocomposite prepared from date palm pits (*Phoenix dactylifera* L.). Int. J. Biol. Macromol..

[157] Pandey S., Makhado E., Kim S., Kang M. (2023). Recent developments of polysaccharide based superabsorbent nanocomposite for organic dye contamination removal from wastewater—A review. Environ. Res..

[158] Salahuddin B., Aziz S., Gao S., Hossain M.S.A., Billah M., Zhu Z., Amiralian N. (2022). Magnetic Hydrogel Composite for Wastewater Treatment. Polymers.

[159] Pereira A.G.B., Rodrigues F.H.A., Paulino A.T., Martins A.F., Fajardo A.R. (2021). Recent advances on composite hydrogels designed for the remediation of dye-contaminated water and wastewater: A review. J. Clean. Prod..

[160] Miao Q., Jiang L., Yang J., Hu T., Shan S., Su H., Wu F. (2022). MOF/hydrogel composite-based adsorbents for water treatment: A review. J. Water Process Eng..

[161] Alsamman M.T., Sánchez J. (2021). Recent advances on hydrogels based on chitosan and alginate for the adsorption of dyes and metal ions from water. Arab. J. Chem..

[162] Doondani P., Jugade R., Gomase V., Shekhawat A., Bambal A., Pandey S. (2022). Chitosan/Graphite/Polyvinyl Alcohol Magnetic Hydrogel Microspheres for Decontamination of Reactive Orange 16 Dye. Water.

[163] Zhang C., Dai Y., Wu Y., Lu G., Cao Z., Cheng J., Wang K., Yang H., Xia Y., Wen X. (2020). Facile preparation of polyacrylamide/chitosan/Fe_3_O_4_ composite hydrogels for effective removal of methylene blue from aqueous solution. Carbohydr. Polym..

[164] Singh N., Riyajuddin S., Ghosh K., Mehta S.K., Dan A. (2019). Chitosan-Graphene Oxide Hydrogels with Embedded Magnetic Iron Oxide Nanoparticles for Dye Removal. ACS Appl. Nano Mater..

[165] Zhao D., Shen Z., Shen X. (2021). Dual-functional calcium alginate hydrogel beads for disinfection control and removal of dyes in water. Int. J. Biol. Macromol..

[166] Sarkar N., Sahoo G., Swain S.K. (2020). Graphene quantum dot decorated magnetic graphene oxide filled polyvinyl alcohol hybrid hydrogel for removal of dye pollutants. J. Mol. Liq..

[167] Hingrajiya R.D., Patel M.P. (2023). Fe_3_O_4_ modified chitosan based co-polymeric magnetic composite hydrogel: Synthesis, characterization and evaluation for the removal of methylene blue from aqueous solutions. Int. J. Biol. Macromol..

[168] Meng Y., Li C., Liu X., Lu J., Cheng Y., Xiao L.-P., Wang H. (2019). Preparation of magnetic hydrogel microspheres of lignin derivate for application in water. Sci. Total Environ..

[169] Sanchez L.M., Shuttleworth P.S., Waiman C., Zanini G., Alvarez V.A., Ollier R.P. (2020). Physically-crosslinked polyvinyl alcohol composite hydrogels containing clays, carbonaceous materials and magnetic nanoparticles as fillers. J. Environ. Chem. Eng..

[170] Facchi D.P., Cazetta A.L., Canesin E.A., Almeida V.C., Bonafé E.G., Kipper M.J., Martins A.F. (2018). New magnetic chitosan/alginate/Fe_3_O_4_@SiO_2_ hydrogel composites applied for removal of Pb(II) ions from aqueous systems. Chem. Eng. J..

[171] Wu S., Guo J., Wang Y., Huang C., Hu Y. (2021). Facile preparation of magnetic sodium alginate/carboxymethyl cellulose composite hydrogel for removal of heavy metal ions from aqueous solution. J. Mater. Sci..

[172] Lv S.-W., Liu J.-M., Wang Z.-H., Ma H., Li C.-Y., Zhao N., Wang S. (2019). Recent advances on porous organic frameworks for the adsorptive removal of hazardous materials. J. Environ. Sci..

[173] Wang C., Liu X., Yang T., Sridhar D., Algadi H., Bin Xu B., El-Bahy Z.M., Li H., Ma Y., Li T. (2023). An overview of metal-organic frameworks and their magnetic composites for the removal of pollutants. Sep. Purif. Technol..

[174] Jarrah A., Farhadi S. (2020). Preparation and characterization of novel polyoxometalate/CoFe_2_O_4_/metal–organic framework magnetic core–shell nanocomposites for the rapid removal of organic dyes from water. RSC Adv..

[175] Paz R., Viltres H., Gupta N.K., Leyva C. (2021). Fabrication of magnetic cerium-organic framework-activated carbon composite for charged dye removal from aqueous solutions. J. Mol. Liq..

[176] Ventura K., Arrieta R.A., Marcos-Hernández M., Jabbari V., Powell C.D., Turley R., Lounsbury A.W., Zimmerman J.B., Gardea-Torresdey J., Wong M.S. (2020). Superparamagnetic MOF@GO Ni and Co based hybrid nanocomposites as efficient water pollutant adsorbents. Sci. Total Environ..

[177] Sajjadi S., Khataee A., Darvishi Cheshmeh Soltani R., Bagheri N., Karimi A., Ebadi Fard Azar A. (2018). Implementation of magnetic Fe_3_O_4_@ZIF-8 nanocomposite to activate sodium percarbonate for highly effective degradation of organic compound in aqueous solution. J. Ind. Eng. Chem..

[178] Yang R., Peng Q., Yu B., Shen Y., Cong H. (2021). Yolk-shell Fe_3_O_4_@MOF-5 nanocomposites as a heterogeneous Fenton-like catalyst for organic dye removal. Sep. Purif. Technol..

[179] Liu Z., He W., Zhang Q., Shapour H., Bakhtari M.F. (2021). Preparation of a GO/MIL-101(Fe) Composite for the Removal of Methyl Orange from Aqueous Solution. ACS Omega.

[180] Wang Y., He L., Li Y., Jing L., Wang J., Li X. (2020). Ag NPs supported on the magnetic Al-MOF/PDA as nanocatalyst for the removal of organic pollutants in water. J. Alloys Compd..

[181] Yang Q., Ren S., Zhao Q., Lu R., Hang C., Chen Z., Zheng H. (2018). Selective separation of methyl orange from water using magnetic ZIF-67 composites. Chem. Eng. J..

[182] Valadi F.M., Ekramipooya A., Gholami M.R. (2020). Selective separation of Congo Red from a mixture of anionic and cationic dyes using magnetic-MOF: Experimental and DFT study. J. Mol. Liq..

[183] Far H.S., Hasanzadeh M., Nashtaei M.S., Rabbani M., Haji A., Hadavi Moghadam B. (2020). PPI-Dendrimer-Functionalized Magnetic Metal–Organic Framework (Fe_3_O_4_@MOF@PPI) with High Adsorption Capacity for Sustainable Wastewater Treatment. ACS Appl. Mater. Interfaces.

[184] Wu G., Ma J., Li S., Guan J., Jiang B., Wang L., Li J., Wang X., Chen L. (2018). Magnetic copper-based metal organic framework as an effective and recyclable adsorbent for removal of two fluoroquinolone antibiotics from aqueous solutions. J. Colloid Interface Sci..

[185] Fan S., Qu Y., Yao L., Ren J., Luque R., He Z., Bai C. (2021). MOF-derived cluster-shaped magnetic nanocomposite with hierarchical pores as an efficient and regenerative adsorbent for chlortetracycline removal. J. Colloid Interface Sci..

[186] Zhao F., Fang S., Gao Y., Bi J. (2022). Removal of aqueous pharmaceuticals by magnetically functionalized Zr-MOFs: Adsorption Kinetics, Isotherms, and regeneration. J. Colloid Interface Sci..

[187] Wang M., Zhao Z., Lin S., Su M., Liang B., Liang S. (2022). New insight into the co-adsorption of oxytetracycline and Pb(II) using magnetic metal–organic frameworks composites in aqueous environment: Co-adsorption mechanisms and application potentials. Environ. Sci. Pollut. Res..

[188] Zheng X., Wang J., Xue X., Liu W., Kong Y., Cheng R., Yuan D. (2018). Facile synthesis of Fe_3_O_4_@MOF-100(Fe) magnetic microspheres for the adsorption of diclofenac sodium in aqueous solution. Environ. Sci. Pollut. Res..

[189] Lu D., Qin M., Liu C., Deng J., Shi G., Zhou T. (2021). Ionic Liquid-Functionalized Magnetic Metal–Organic Framework Nanocomposites for Efficient Extraction and Sensitive Detection of Fluoroquinolone Antibiotics in Environmental Water. ACS Appl. Mater. Interfaces.

[190] Yuan G., Zhao C., Tu H., Li M., Liu J., Liao J., Yang Y., Yang J., Liu N. (2018). Removal of Co(II) from aqueous solution with Zr-based magnetic metal-organic framework composite. Inorganica Chim. Acta.

[191] Omer A.M., Abd El-Monaem E.M., Abd El-Latif M.M., El-Subruiti G.M., Eltaweil A.S. (2021). Facile fabrication of novel magnetic ZIF-67 MOF@aminated chitosan composite beads for the adsorptive removal of Cr(VI) from aqueous solutions. Carbohydr. Polym..

[192] Wang C., Xiong C., He Y., Yang C., Li X., Zheng J., Wang S. (2021). Facile preparation of magnetic Zr-MOF for adsorption of Pb(II) and Cr(VI) from water: Adsorption characteristics and mechanisms. Chem. Eng. J..

[193] Li L., Xu Y., Zhong D., Zhong N. (2020). CTAB-surface-functionalized magnetic MOF@MOF composite adsorbent for Cr(VI) efficient removal from aqueous solution. Colloids Surf. A Physicochem. Eng. Asp..

[194] Prakash Tripathy S., Subudhi S., Das S., Kumar Ghosh M., Das M., Acharya R., Acharya R., Parida K. (2022). Hydrolytically stable citrate capped Fe_3_O_4_@UiO-66-NH_2_ MOF: A hetero-structure composite with enhanced activity towards Cr (VI) adsorption and photocatalytic H_2_ evolution. J. Colloid Interface Sci..

[195] Zhou T., Liang Q., Zhou X., Luo H., Chen W. (2021). Enhanced removal of toxic hexavalent chromium from aqueous solution by magnetic Zr-MOF@polypyrrole: Performance and mechanism. Environ. Sci. Pollut. Res..

[196] Huo J.-B., Xu L., Chen X., Zhang Y., Yang J.-C.E., Yuan B., Fu M.-L. (2019). Direct epitaxial synthesis of magnetic Fe_3_O_4_@UiO-66 composite for efficient removal of arsenate from water. Microporous Mesoporous Mater..

[197] Li J., Lin G., Tan F., Fu L., Zeng B., Wang S., Hu T., Zhang L. (2023). Selective adsorption of mercury ion from water by a novel functionalized magnetic Ti based metal-organic framework composite. J. Colloid Interface Sci..

[198] Li Y., Tan M., Liu G., Si D., Chen N., Zhou D. (2022). Thiol-functionalized metal–organic frameworks embedded with chelator-modified magnetite for high-efficiency and recyclable mercury removal in aqueous solutions. J. Mater. Chem. A.

[199] Jiang X., Su S., Rao J., Li S., Lei T., Bai H., Wang S., Yang X. (2021). Magnetic metal-organic framework (Fe_3_O_4_@ZIF-8) core-shell composite for the efficient removal of Pb(II) and Cu(II) from water. J. Environ. Chem. Eng..

[200] Goyal P., Tiwary C.S., Misra S.K. (2021). Ion exchange based approach for rapid and selective Pb(II) removal using iron oxide decorated metal organic framework hybrid. J. Environ. Manag..

[201] Wang Z., Zhao D., Wu C., Chen S., Wang Y., Chen C. (2020). Magnetic metal organic frameworks/graphene oxide adsorbent for the removal of U(VI) from aqueous solution. Appl. Radiat. Isot..

[202] Li W.-T., Shi W., Hu Z.-J., Yang T., Chen M.-L., Zhao B., Wang J.-H. (2020). Fabrication of magnetic Fe_3_O_4_@metal organic framework@covalent organic framework composite and its selective separation of trace copper. Appl. Surf. Sci..

[203] Bui T.T., Nguyen D.C., Hua S.H., Chun H., Kim Y.S. (2022). Sonochemical Preparation of a Magnet-Responsive Fe_3_O_4_@ZIF-8 Adsorbent for Efficient Cu^2+^ Removal. Nanomaterials.

[204] Fan L., Deng M., Lin C., Xu C., Liu Y., Shi Z., Wang Y., Xu Z., Li L., He M. (2018). A multifunctional composite Fe_3_O_4_/MOF/l-cysteine for removal, magnetic solid phase extraction and fluorescence sensing of Cd(ii). RSC Adv..

[205] Yang J., Huang L., You J., Yamauchi Y. (2023). Magnetic Covalent Organic Framework Composites for Wastewater Remediation. Small.

[206] Li Y., Zhang H., Chen Y., Huang L., Lin Z., Cai Z. (2019). Core–Shell Structured Magnetic Covalent Organic Framework Nanocomposites for Triclosan and Triclocarban Adsorption. ACS Appl. Mater. Interfaces.

[207] He S., Zeng T., Wang S., Niu H., Cai Y. (2017). Facile Synthesis of Magnetic Covalent Organic Framework with Three-Dimensional Bouquet-Like Structure for Enhanced Extraction of Organic Targets. ACS Appl. Mater. Interfaces.

[208] Li Y., Yang C.-X., Yan X.-P. (2017). Controllable preparation of core–shell magnetic covalent-organic framework nanospheres for efficient adsorption and removal of bisphenols in aqueous solution. Chem. Commun..

[209] Zhong X., Lu Z., Liang W., Hu B. (2020). The magnetic covalent organic framework as a platform for high-performance extraction of Cr(VI) and bisphenol a from aqueous solution. J. Hazard. Mater..

[210] Zhang W., Liang F., Li C., Qiu L.-G., Yuan Y.-P., Peng F.-M., Jiang X., Xie A.-J., Shen Y.-H., Zhu J.-F. (2011). Microwave-enhanced synthesis of magnetic porous covalent triazine-based framework composites for fast separation of organic dye from aqueous solution. J. Hazard. Mater..

[211] Zhuang S., Chen R., Liu Y., Wang J. (2020). Magnetic COFs for the adsorptive removal of diclofenac and sulfamethazine from aqueous solution: Adsorption kinetics, isotherms study and DFT calculation. J. Hazard. Mater..

[212] Shen H., Chen L., Zhou C., Du J., Lu C., Yang H., Tan L., Zeng X., Dong L. (2022). Immobilizing Fe^0^ nanoparticles on covalent organic framework towards enhancement of Cr(VI) removal by adsorption and reduction synergistic effect. Sep. Purif. Technol..

[213] Wang S., Wang H., Wang S., Fu L., Zhang L. (2023). Novel magnetic covalent organic framework for the selective and effective removal of hazardous metal Pb(II) from solution: Synthesis and adsorption characteristics. Sep. Purif. Technol..

[214] Huang L., Shen R., Liu R., Shuai Q. (2020). Thiol-functionalized magnetic covalent organic frameworks by a cutting strategy for efficient removal of Hg^2+^ from water. J. Hazard. Mater..

[215] Ge J., Xiao J., Liu L., Qiu L., Jiang X. (2016). Facile microwave-assisted production of Fe_3_O_4_ decorated porous melamine-based covalent organic framework for highly selective removal of Hg^2+^. J. Porous Mater..

[216] Bai Y., Yang J., Shuai Q., Huang L. (2023). Highly efficiency and selective recovery of gold using magnetic covalent organic framework through synergistic adsorption and reduction. Colloids Surf. A Physicochem. Eng. Asp..

[217] Zhong X., Lu Z., Liang W., Hu B. (2021). Incorporating bimetal oxide MnFe_2_O_4_ onto covalent organic frameworks for the removal of UO_2_^2^+ ion from aqueous solution. Appl. Surf. Sci..

[218] Tirado-Guizar A., González-Gómez W., Pina-Luis G., Galindo J.T.E., Paraguay-Delgado F. (2020). Anthracene removal from water samples using a composite based on metal-organic-frameworks (MIL-101) and magnetic nanoparticles (Fe_3_O_4_). Nanotechnology.

[219] Senosy I.A., Lu Z.-H., Abdelrahman T.M., Yang M.-N.O., Guo H.-M., Yang Z.-H., Li J.-H. (2020). The post-modification of magnetic metal–organic frameworks with β-cyclodextrin for the efficient removal of fungicides from environmental water. Environ. Sci. Nano.

[220] Li Y., Zhou X., Dong L., Lai Y., Li S., Liu R., Liu J. (2019). Magnetic metal-organic frameworks nanocomposites for negligible-depletion solid-phase extraction of freely dissolved polyaromatic hydrocarbons. Environ. Pollut..

[221] Chen M., Wang N., Wang X., Zhou Y., Zhu L. (2021). Enhanced degradation of tetrabromobisphenol A by magnetic Fe_3_O_4_@ZIF-67 composites as a heterogeneous Fenton-like catalyst. Chem. Eng. J..

[222] Xue Y., Xiang P., Jiang Y., Lian H., Mo J., Li M. (2020). Influence of Ca^2+^ on phosphate removal from water using a non-core-shell Fe_3_O_4_@ZIF-67 composites. J. Environ. Chem. Eng..

[223] Jadhav S.A., Garud H.B., Patil A.H., Patil G.D., Patil C.R., Dongale T.D., Patil P.S. (2019). Recent advancements in silica nanoparticles based technologies for removal of dyes from water. Colloid Interface Sci. Commun..

[224] Cendrowski K., Sikora P., Zielinska B., Horszczaruk E., Mijowska E. (2017). Chemical and thermal stability of core-shelled magnetite nanoparticles and solid silica. Appl. Surf. Sci..

[225] Diagboya P.N.E., Dikio E.D. (2018). Silica-based mesoporous materials; emerging designer adsorbents for aqueous pollutants removal and water treatment. Microporous Mesoporous Mater..

[226] Salman M., Jahan S., Kanwal S., Mansoor F. (2019). Recent advances in the application of silica nanostructures for highly improved water treatment: A review. Environ. Sci. Pollut. Res..

[227] Wang L., Shen C., Cao Y. (2019). PVP modified Fe_3_O_4_@SiO_2_ nanoparticles as a new adsorbent for hydrophobic substances. J. Phys. Chem. Solids.

[228] Chen J., Chen H. (2018). Removal of anionic dyes from an aqueous solution by a magnetic cationic adsorbent modified with DMDAAC. New J. Chem..

[229] Jiaqi Z., Yimin D., Danyang L., Shengyun W., Liling Z., Yi Z. (2019). Synthesis of carboxyl-functionalized magnetic nanoparticle for the removal of methylene blue. Colloids Surf. A Physicochem. Eng. Asp..

[230] Wo R., Li Q.-L., Zhu C., Zhang Y., Qiao G., Lei K., Du P., Jiang W. (2019). Preparation and Characterization of Functionalized Metal–Organic Frameworks with Core/Shell Magnetic Particles (Fe_3_O_4_@SiO_2_@MOFs) for Removal of Congo Red and Methylene Blue from Water Solution. J. Chem. Eng. Data.

[231] Oppmann M., Wozar M., Reichstein J., Mandel K. (2019). Reusable Superparamagnetic Raspberry-Like Supraparticle Adsorbers as Instant Cleaning Agents for Ultrafast Dye Removal from Water. ChemNanoMat.

[232] Zhao J., Li W., Fan L., Quan Q., Wang J., Xiao C. (2019). Yolk-porous shell nanospheres from siliver-decorated titanium dioxide and silicon dioxide as an enhanced visible-light photocatalyst with guaranteed shielding for organic carrier. J. Colloid Interface Sci..

[233] Yang Q., Wang J., Chen X., Yang W., Pei H., Hu N., Li Z., Suo Y., Li T., Wang J. (2018). The simultaneous detection and removal of organophosphorus pesticides by a novel Zr-MOF based smart adsorbent. J. Mater. Chem. A.

[234] Ghorbani F., Kamari S. (2019). Core–shell magnetic nanocomposite of Fe_3_O_4_@SiO_2_@NH_2_ as an efficient and highly recyclable adsorbent of methyl red dye from aqueous environments. Environ. Technol. Innov..

[235] Zhang Y., Jiang F., Huang D., Hou S., Wang H., Wang M., Chi Y., Zhao Z. (2018). A facile route to magnetic mesoporous core–shell structured silicas containing covalently bound cyclodextrins for the removal of the antibiotic doxycycline from water. RSC Adv..

[236] Ghanei M., Rashidi A., Tayebi H.-A., Yazdanshenas M.E. (2018). Removal of Acid Blue 25 from Aqueous Media by Magnetic-SBA-15/CPAA Super Adsorbent: Adsorption Isotherm, Kinetic, and Thermodynamic Studies. J. Chem. Eng. Data.

[237] Zhang M., Yang J., Geng X., Li Y., Zha Z., Cui S., Yang J. (2019). Magnetic adsorbent based on mesoporous silica nanoparticles for magnetic solid phase extraction of pyrethroid pesticides in water samples. J. Chromatogr. A.

[238] He J., Zeng X., Lan S., Lo I.M.C. (2019). Reusable magnetic Ag/Fe, N-TiO_2_/Fe_3_O_4_@SiO_2_ composite for simultaneous photocatalytic disinfection of *E. coli* and degradation of bisphenol A in sewage under visible light. Chemosphere.

[239] Xiao Y., Deng Y., Huan W., Li J., Zhang J., Xing M. (2019). Hollow-structured Fe_2_O_3_/Au/SiO_2_ nanorods with enhanced and recyclable photo-Fenton oxidation for the remediation of organic pollutants. Mater. Today Chem..

[240] Wang J., Tong X., Chen Y., Sun T., Liang L., Wang C. (2020). Enhanced removal of Cr(III) in high salt organic wastewater by EDTA modified magnetic mesoporous silica. Microporous Mesoporous Mater..

[241] Nicola R., Costişor O., Ciopec M., Negrea A., Lazău R., Ianăşi C., Picioruş E.-M., Len A., Almásy L., Szerb E.I. (2020). Silica-Coated Magnetic Nanocomposites for Pb2+ Removal from Aqueous Solution. Appl. Sci..

[242] Ganesamoorthy R., Vadivel V.K., Kumar R., Kushwaha O.S., Mamane H. (2021). Aerogels for water treatment: A review. J. Clean. Prod..

[243] Peng H., Xiong W., Yang Z., Xu Z., Cao J., Jia M., Xiang Y. (2022). Advanced MOFs@aerogel composites: Construction and application towards environmental remediation. J. Hazard. Mater..

[244] Shah N., Rehan T., Li X., Tetik H., Yang G., Zhao K., Lin D. (2021). Magnetic aerogel: An advanced material of high importance. RSC Adv..

[245] Krishna Kumar A.S., Warchol J., Matusik J., Tseng W.-L., Rajesh N., Bajda T. (2022). Heavy metal and organic dye removal via a hybrid porous hexagonal boron nitride-based magnetic aerogel. npj Clean Water.

[246] Lei C., Wen F., Chen J., Chen W., Huang Y., Wang B. (2021). Mussel-inspired synthesis of magnetic carboxymethyl chitosan aerogel for removal cationic and anionic dyes from aqueous solution. Polymer.

[247] Wang C., Ma R., Huang Z., Liu X., Wang T., Chen K. (2021). Preparation and characterization of carboxymethylcellulose based citric acid cross-linked magnetic aerogel as an efficient dye adsorbent. Int. J. Biol. Macromol..

[248] Ali I., Neskoromnaya E.A., Melezhik A.V., Babkin A.V., Kulnitskiy B.A., Burakov A.E., Burakova I.V., Tkachev A.G., Almalki A.S.A., Alsubaie A. (2022). Magnetically active nanocomposite aerogels: Preparation, characterization and application for water treatment. J. Porous Mater..

[249] Xiong J., Zhang D., Lin H., Chen Y. (2020). Amphiprotic cellulose mediated graphene oxide magnetic aerogels for water remediation. Chem. Eng. J..

[250] Zhai S., Chen R., Liu J., Xu J., Jiang H. (2021). N-doped magnetic carbon aerogel for the efficient adsorption of Congo red. J. Taiwan Inst. Chem. Eng..

[251] Tang R., Hong W., Srinivasakannan C., Liu X., Wang X., Duan X. (2022). A novel mesoporous Fe-silica aerogel composite with phenomenal adsorption capacity for malachite green. Sep. Purif. Technol..

[252] Arabkhani P., Asfaram A. (2020). Development of a novel three-dimensional magnetic polymer aerogel as an efficient adsorbent for malachite green removal. J. Hazard. Mater..

[253] Bober P., Minisy I.M., Acharya U., Pfleger J., Babayan V., Kazantseva N., Hodan J., Stejskal J. (2020). Conducting polymer composite aerogel with magnetic properties for organic dye removal. Synth. Met..

[254] Cheng Y., Cai Y., Wang Z., Lu X., Xia H. (2022). Ultralight NiCo@rGO aerogel microspheres with magnetic response for oil/water separation. Chem. Eng. J..

[255] Li Y., Zhou M., Waterhouse G.I.N., Sun J., Shi W., Ai S. (2021). Efficient removal of cadmium ions from water by adsorption on a magnetic carbon aerogel. Environ. Sci. Pollut. Res..

[256] Kang W., Cui Y., Qin L., Yang Y., Zhao Z., Wang X., Liu X. (2020). A novel robust adsorbent for efficient oil/water separation: Magnetic carbon nanospheres/graphene composite aerogel. J. Hazard. Mater..

[257] Deem L.M., Crow S.E. (2017). Biochar. Reference Module in Earth Systems and Environmental Sciences.

[258] Yadav N.K., Singh S.K., Patel A.B., Meitei M.M., Meena D.K., Yadav M.K., Lal J., Choudhary B.K., Sarathchandran M.R.U., Thomas S., Meena D.K. (2023). Chapter 16—Biochar production methods vis-a-vis aquaculture applications: A strategy for sustainable paradigm. Organic Farming.

[259] Bushra B., Remya N. (2020). Biochar from pyrolysis of rice husk biomass—Characteristics, modification and environmental application. Biomass Convers. Biorefinery.

[260] Premarathna K.S.D., Rajapaksha A.U., Sarkar B., Kwon E.E., Bhatnagar A., Ok Y.S., Vithanage M. (2019). Biochar-based engineered composites for sorptive decontamination of water: A review. Chem. Eng. J..

[261] Agasti N. (2021). Decontamination of heavy metal ions from water by composites prepared from waste. Curr. Res. Green Sustain. Chem..

[262] Li X., Wang C., Zhang J., Liu J., Liu B., Chen G. (2020). Preparation and application of magnetic biochar in water treatment: A critical review. Sci. Total Environ..

[263] Zeng H., Qi W., Zhai L., Wang F., Zhang J., Li D. (2021). Preparation and Characterization of Sludge-Based Magnetic Biochar by Pyrolysis for Methylene Blue Removal. Nanomaterials.

[264] Yao X., Ji L., Guo J., Ge S., Lu W., Cai L., Wang Y., Song W., Zhang H. (2020). Magnetic activated biochar nanocomposites derived from wakame and its application in methylene blue adsorption. Bioresour. Technol..

[265] Luyen N.T., Linh H.X., Huy T.Q. (2020). Preparation of Rice Husk Biochar-Based Magnetic Nanocomposite for Effective Removal of Crystal Violet. J. Electron. Mater..

[266] Liang G., Wang Z., Yang X., Qin T., Xie X., Zhao J., Li S. (2019). Efficient removal of oxytetracycline from aqueous solution using magnetic montmorillonite-biochar composite prepared by one step pyrolysis. Sci. Total Environ..

[267] Silva C.P., Pereira D., Calisto V., Martins M.A., Otero M., Esteves V.I., Lima D.L.D. (2021). Biochar-TiO_2_ magnetic nanocomposites for photocatalytic solar-driven removal of antibiotics from aquaculture effluents. J. Environ. Manag..

[268] Sun H., Yang J., Wang Y., Liu Y., Cai C., Davarpanah A. (2021). Study on the Removal Efficiency and Mechanism of Tetracycline in Water Using Biochar and Magnetic Biochar. Coatings.

[269] You Y., Shi Z., Li Y., Zhao Z., He B., Cheng X. (2021). Magnetic cobalt ferrite biochar composite as peroxymonosulfate activator for removal of lomefloxacin hydrochloride. Sep. Purif. Technol..

[270] Heo J., Yoon Y., Lee G., Kim Y., Han J., Park C.M. (2019). Enhanced adsorption of bisphenol A and sulfamethoxazole by a novel magnetic CuZnFe_2_O_4_–biochar composite. Bioresour. Technol..

[271] Guo Z., Chen R., Yang R., Yang F., Chen J., Li Y., Zhou R., Xu J. (2020). Synthesis of amino-functionalized biochar/spinel ferrite magnetic composites for low-cost and efficient elimination of Ni(II) from wastewater. Sci. Total Environ..

[272] Zhang Y., Shi G., Wu W., Ali A., Wang H., Wang Q., Xu Z., Qi W., Li R., Zhang Z. (2022). Magnetic biochar composite decorated with amino-containing biopolymer for phosphorus recovery from swine wastewater. Colloids Surf. A Physicochem. Eng. Asp..

[273] Wang S., Xiao D., Zheng X., Zheng L., Yang Y., Zhang H., Ai B., Sheng Z. (2021). Halloysite and coconut shell biochar magnetic composites for the sorption of Pb(II) in wastewater: Synthesis, characterization and mechanism investigation. J. Environ. Chem. Eng..

[274] Zheng Z., Duan X., Tie J. (2022). One-pot synthesis of a magnetic Zn/iron-based sludge/biochar composite for aqueous Cr(VI) adsorption. Environ. Technol. Innov..

[275] Wang X., Xu J., Liu J., Liu J., Xia F., Wang C., Dahlgren R.A., Liu W. (2020). Mechanism of Cr(VI) removal by magnetic greigite/biochar composites. Sci. Total Environ..

[276] Li M., Liu H., Chen T., Dong C., Sun Y. (2019). Synthesis of magnetic biochar composites for enhanced uranium(VI) adsorption. Sci. Total Environ..

[277] Pan J., Gao B., Wang S., Guo K., Xu X., Yue Q. (2020). Waste-to-resources: Green preparation of magnetic biogas residues-based biochar for effective heavy metal removals. Sci. Total Environ..

[278] Qin L., He L., Yang W., Lin A. (2020). Preparation of a novel iron-based biochar composite for removal of hexavalent chromium in water. Environ. Sci. Pollut. Res..

[279] Ayalew A.A. (2022). A critical review on clay-based nanocomposite particles for application of wastewater treatment. Water Sci. Technol..

[280] Fadillah G., Yudha S.P., Sagadevan S., Fatimah I., Muraza O. (2020). Magnetic iron oxide/clay nanocomposites for adsorption and catalytic oxidation in water treatment applications. Open Chem..

[281] Han H., Rafiq M.K., Zhou T., Xu R., Mašek O., Li X. (2019). A critical review of clay-based composites with enhanced adsorption performance for metal and organic pollutants. J. Hazard. Mater..

[282] Magdy A., Fouad Y.O., Abdel-Aziz M.H., Konsowa A.H. (2017). Synthesis and characterization of Fe_3_O_4_/kaolin magnetic nanocomposite and its application in wastewater treatment. J. Ind. Eng. Chem..

[283] Sanad M.M.S., Farahat M.M., Abdel Khalek M.A. (2021). One-step processing of low-cost and superb natural magnetic adsorbent: Kinetics and thermodynamics investigation for dye removal from textile wastewater. Adv. Powder Technol..

[284] Chen X., Cui J., Xu X., Sun B., Zhang L., Dong W., Chen C., Sun D. (2020). Bacterial cellulose/attapulgite magnetic composites as an efficient adsorbent for heavy metal ions and dye treatment. Carbohydr. Polym..

[285] Diagboya P.N., Dikio E.D. (2018). Scavenging of aqueous toxic organic and inorganic cations using novel facile magneto-carbon black-clay composite adsorbent. J. Clean. Prod..

[286] Tran N.B.T., Duong N.B., Le N.L. (2021). Synthesis and characterization of magnetic Fe_3_O_4_/zeolite NaA nanocomposite for the adsorption removal of methylene blue potential in wastewater treatment. J. Chem..

[287] Zhang B., Zhang T., Zhang Z., Xie M. (2019). Hydrothermal synthesis of a graphene/magnetite/montmorillonite nanocomposite and its ultrasonically assisted methylene blue adsorption. J. Mater. Sci..

[288] Esvandi Z., Foroutan R., Peighambardoust S.J., Akbari A., Ramavandi B. (2020). Uptake of anionic and cationic dyes from water using natural clay and clay/starch/MnFe_2_O_4_ magnetic nanocomposite. Surf. Interfaces.

[289] Karelius K., Sadiana I.M., Fatah A.H., Agnestisia R. (2022). Co-Precipitation Synthesis of Clay-Magnetite Nanocomposite for Adsorptive Removal of Synthetic Dye in Wastewater of Benang Bintik Batik. Molekul.

[290] Peng G., Li T., Ai B., Yang S., Fu J., He Q., Yu G., Deng S. (2019). Highly efficient removal of enrofloxacin by magnetic montmorillonite via adsorption and persulfate oxidation. Chem. Eng. J..

[291] Liu K., Tong Z., Muhammad Y., Huang G., Zhang H., Wang Z., Zhu Y., Tang R. (2020). Synthesis of sodium dodecyl sulfate modified BiOBr/magnetic bentonite photocatalyst with Three-dimensional parterre like structure for the enhanced photodegradation of tetracycline and ciprofloxacin. Chem. Eng. J..

[292] Balbino T.A.C., Bellato C.R., da Silva A.D., Marques Neto J.d.O., Ferreira S.O. (2020). Preparation and evaluation of iron oxide/hydrotalcite intercalated with dodecylsulfate/β-cyclodextrin magnetic organocomposite for phenolic compounds removal. Appl. Clay Sci..

[293] Zong S., Xu X., Ran G., Liu J. (2020). Comparative study of atrazine degradation by magnetic clay activated persulfate and H_2_O_2_. RSC Adv..

[294] Xu X., Chen W., Zong S., Ren X., Liu D. (2019). Magnetic clay as catalyst applied to organics degradation in a combined adsorption and Fenton-like process. Chem. Eng. J..

[295] Sun P., Zhang W., Zou B., Zhou L., Ye Z., Zhao Q. (2021). Preparation of EDTA-modified magnetic attapulgite chitosan gel bead adsorbent for the removal of Cu(II), Pb(II), and Ni(II). Int. J. Biol. Macromol..

[296] Zhang H., Omer A.M., Hu Z., Yang L.-Y., Ji C., Ouyang X. (2019). Fabrication of magnetic bentonite/carboxymethyl chitosan/sodium alginate hydrogel beads for Cu (II) adsorption. Int. J. Biol. Macromol..

[297] Annan E., Nyankson E., Agyei-Tuffour B., Armah S.K., Nkrumah-Buandoh G., Hodasi J.A.M., Oteng-Peprah M. (2021). Synthesis and characterization of modified kaolin-bentonite composites for enhanced fluoride removal from drinking water. Adv. Mater. Sci. Eng..

[298] Choi J.-W., Lee H.-K., Choi S.-J. (2021). Magnetite double-network composite using hydroxyapatite-manganese dioxide for Sr^2+^ removal from aqueous solutions. J. Environ. Chem. Eng..

[299] Shao B., Xu Y., Liu Z., Wu T., Pan Y., Zhang X., He M., Ge L., Lu Y., Liu Y. (2023). Application of carbon aerogel-based materials in persulfate activation for water treatment: A review. J. Clean. Prod..

